# Supplementary material for: ADAPTABLE: a comprehensive web platform of antimicrobial peptides tailored to the user’s research
Source: Life Sci Alliance. 2019 Nov 18;2(6):e201900512. doi: 10.26508/lsa.201900512 (PMC6864362; doi:10.26508/lsa.201900512)
Supplement: Supplementary file 1 [file LSA-2019-00512_Supplemental_Data_1.docx]

# ADAPTABLE

# Tutorial

Content

[ADAPTABLE TUTORIAL – BROWSING INFORMATION ON PEPTIDE SEQUENCES 3](#_Toc517190397)

[How to easily retrieve information from external databases and from the ADAPTABLE family database 3](#_Toc517190398)

[ADAPTABLE TUTORIAL - CASE EXAMPLE n.1 7](#_Toc517190399)

[Designing new peptides active towards a specific organism and highlighting motifs 7](#_Toc517190400)

[The case of *Shigella* 7](#_Toc517190401)

[ADAPTABLE TUTORIAL - CASE EXAMPLE n.2 22](#_Toc517190402)

[Predicting the antimicrobial or anticancer activity of a generic peptide sequence 22](#_Toc517190403)

[The case of random new peptides 22](#_Toc517190404)

[ADAPTABLE TUTORIAL - CASE EXAMPLE n.3 30](#_Toc517190405)

[Discovering new activities of pre-existing sequences not tested experimentally 30](#_Toc517190406)

[The case of *Acinetobacter baumannii* 30](#_Toc517190407)

# ADAPTABLE TUTORIAL – BROWSING INFORMATION ON PEPTIDE SEQUENCES

## How to easily retrieve combined information from external databases and from the ADAPTABLE families database

Suppose you want to retrieve information on a Temporin peptide, whose sequence is FLPIVAKLLSGLL. Go to the “BROWSE AMPs & FAMILIES” page and start to type the name (or part of the sequence) in the dedicated box. While typing, all available entries containing the sequence will be displayed.

***Screenshot 1.***


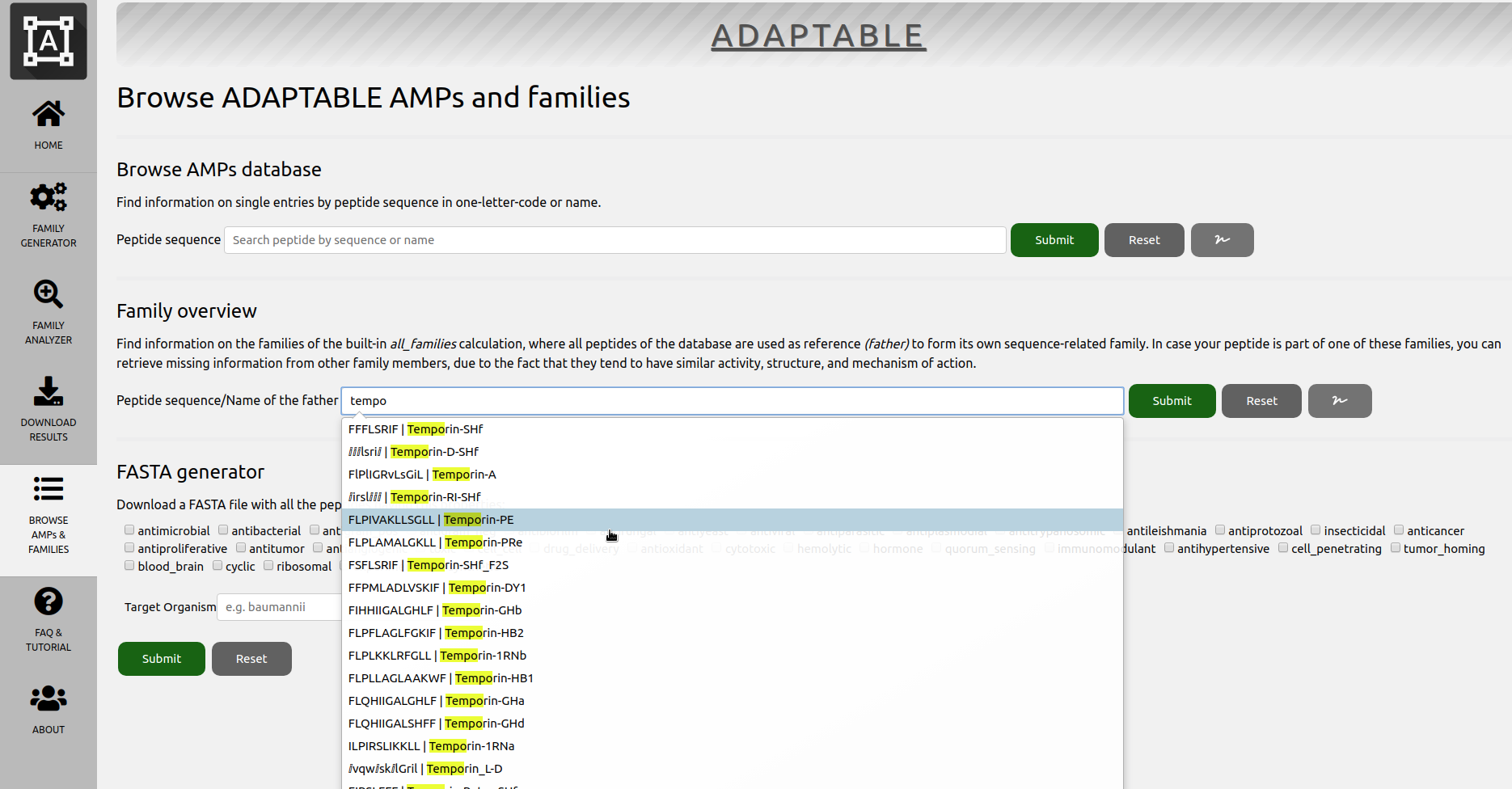


Just click on the desired peptide (the fifth in *screenshot 1*) and click “Submit”. You will see a table as shown in *screenshot 2*.

In the first lines of the table, we learn that your peptide is able to generate a family similar to it (family 2044) but is also a member of other families of the built-in ADAPTABLE families database (f653, f7918, f1651…) that can be also accessed clicking the provided links.

In the field “External database ID” you can see that it has entries in the DBASSP and APD databases (just click on the links to view the related pages of each database). The complete set of information is gathered in the remaining part of the table. We can see if the family generated by our peptide (family 5092) can provide more information related with properties that are still not known for our peptide.


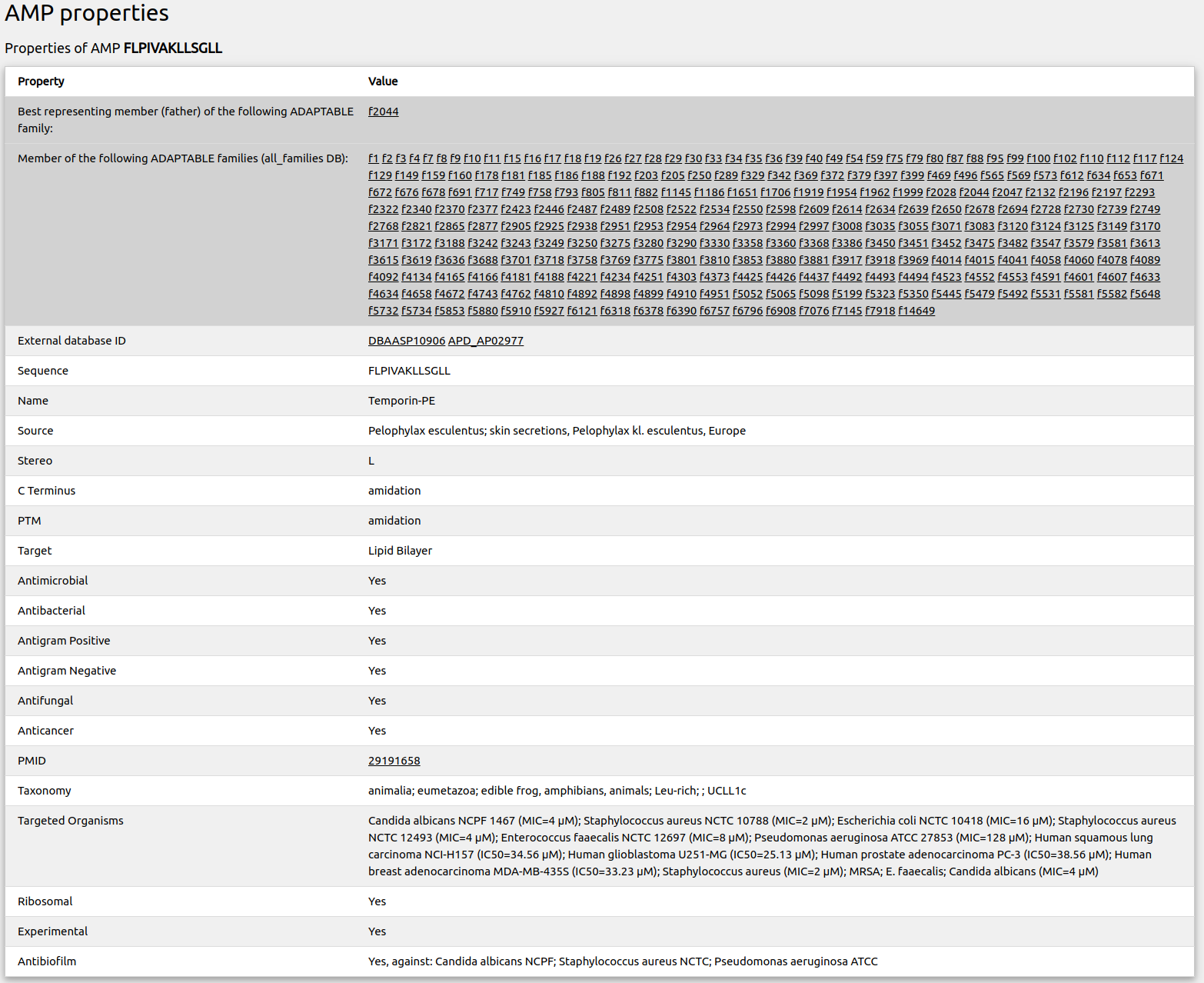
***Screenshot 2***

Clicking on f2044 (first line of the table), or typing the sequence of the peptide used as reference in “BROWSE AMPs & FAMILIES” section (“Family overview” subsection), we are redirected to a page (see *screenshot 3*) describing the characteristics of the family constituted by similar peptides (peptides that can be considered as generated by our sequence, with few amino acid modifications).

The page also shows the list of members of the families (*screenshot 4*) with links to the entries of external databases. From the table in *screenshot 3* we discover that antibacterial data are available for over 78% of the members of the family and that indeed our peptide might display antimicrobial activity. We also learn that most of the similar peptides have a wide range of activities including antifungal but they also can be toxic and hemolytic. The button “Show frequency of aminoacids” allows you to review the members of the family highlighting the frequencies of their aminoacids to make motifs more easily viewable.

In conclusion, the ADAPTABLE family database can suggest properties that have never been tested for some specific entries.

***
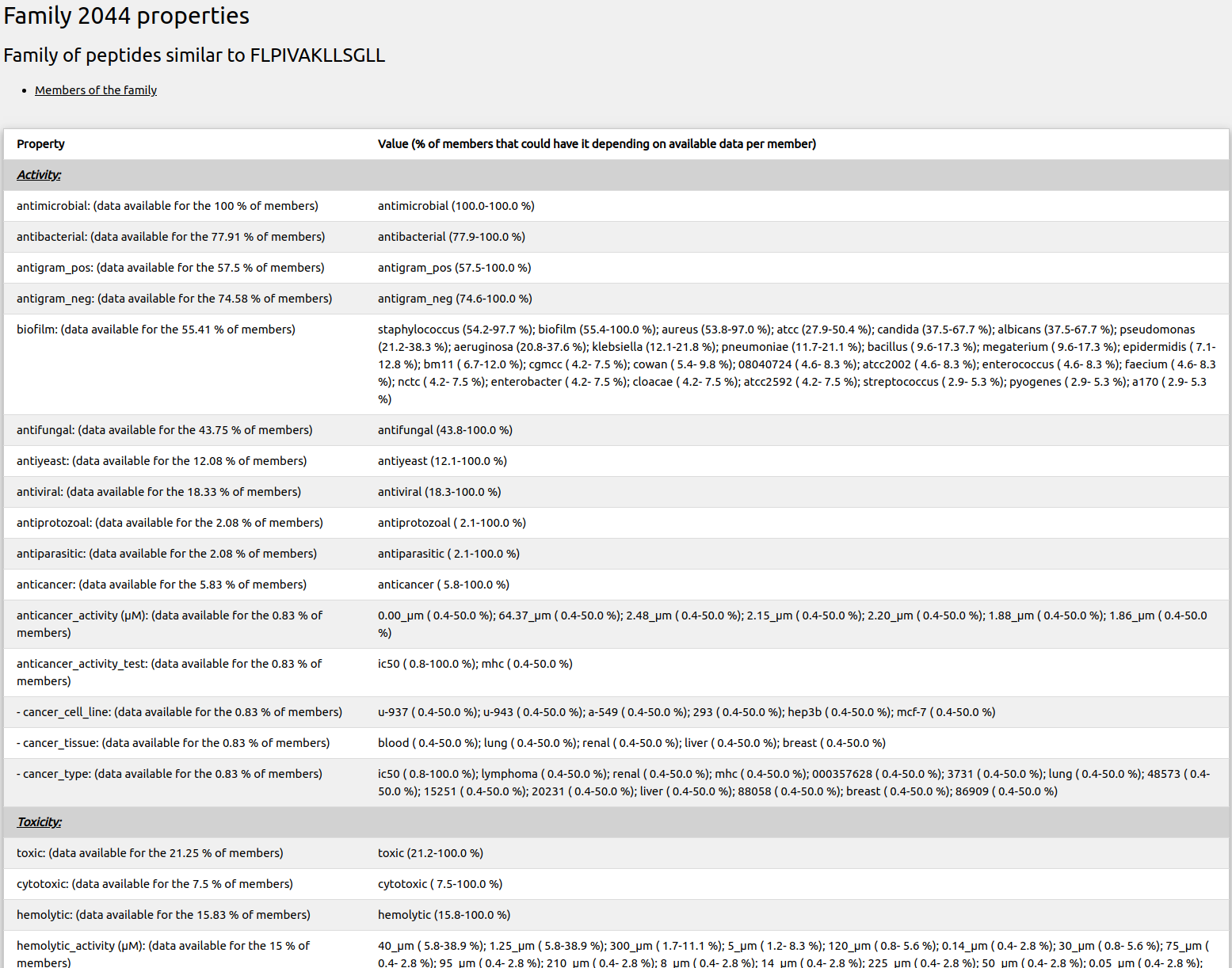
Screenshot3.***

***
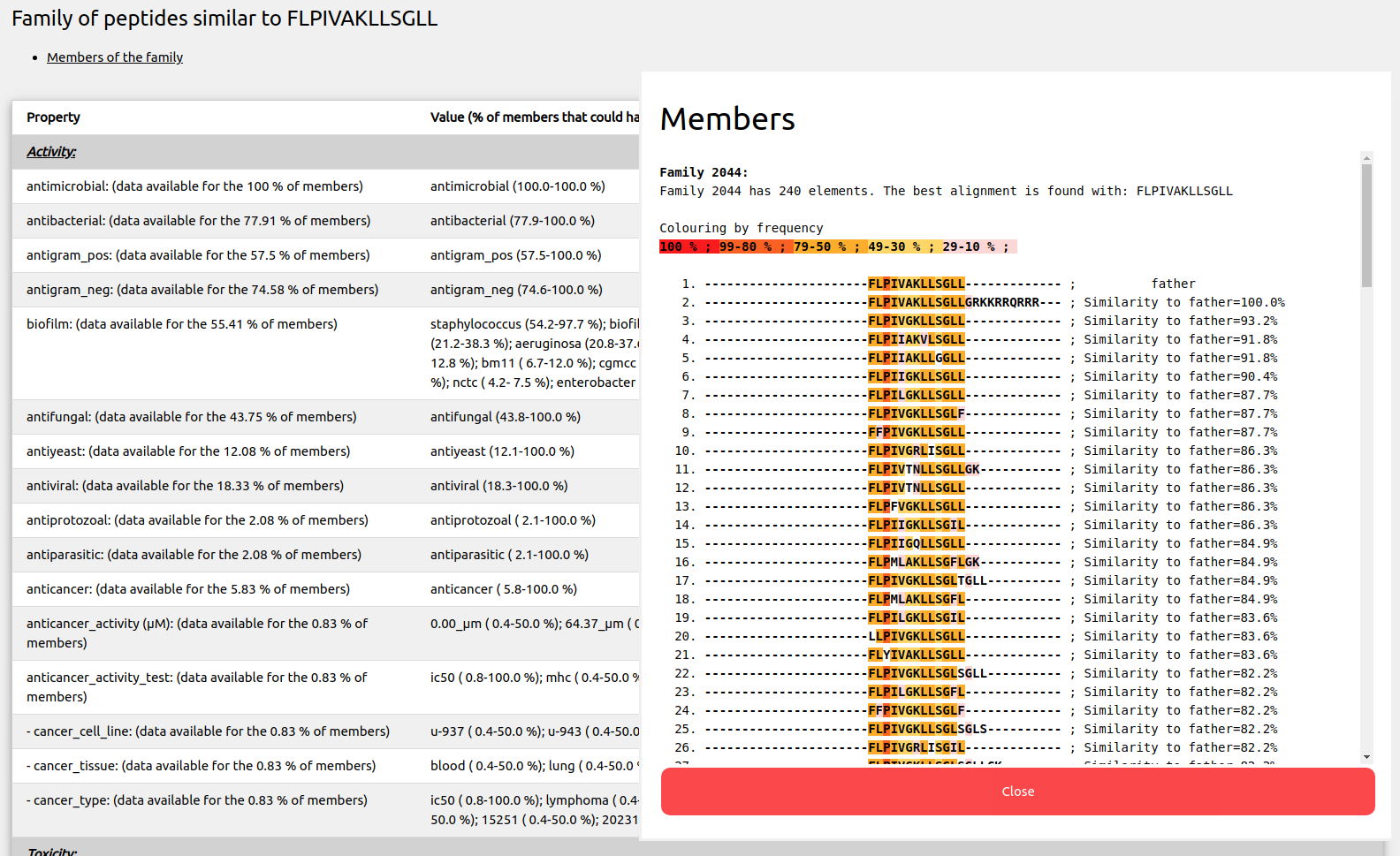
***


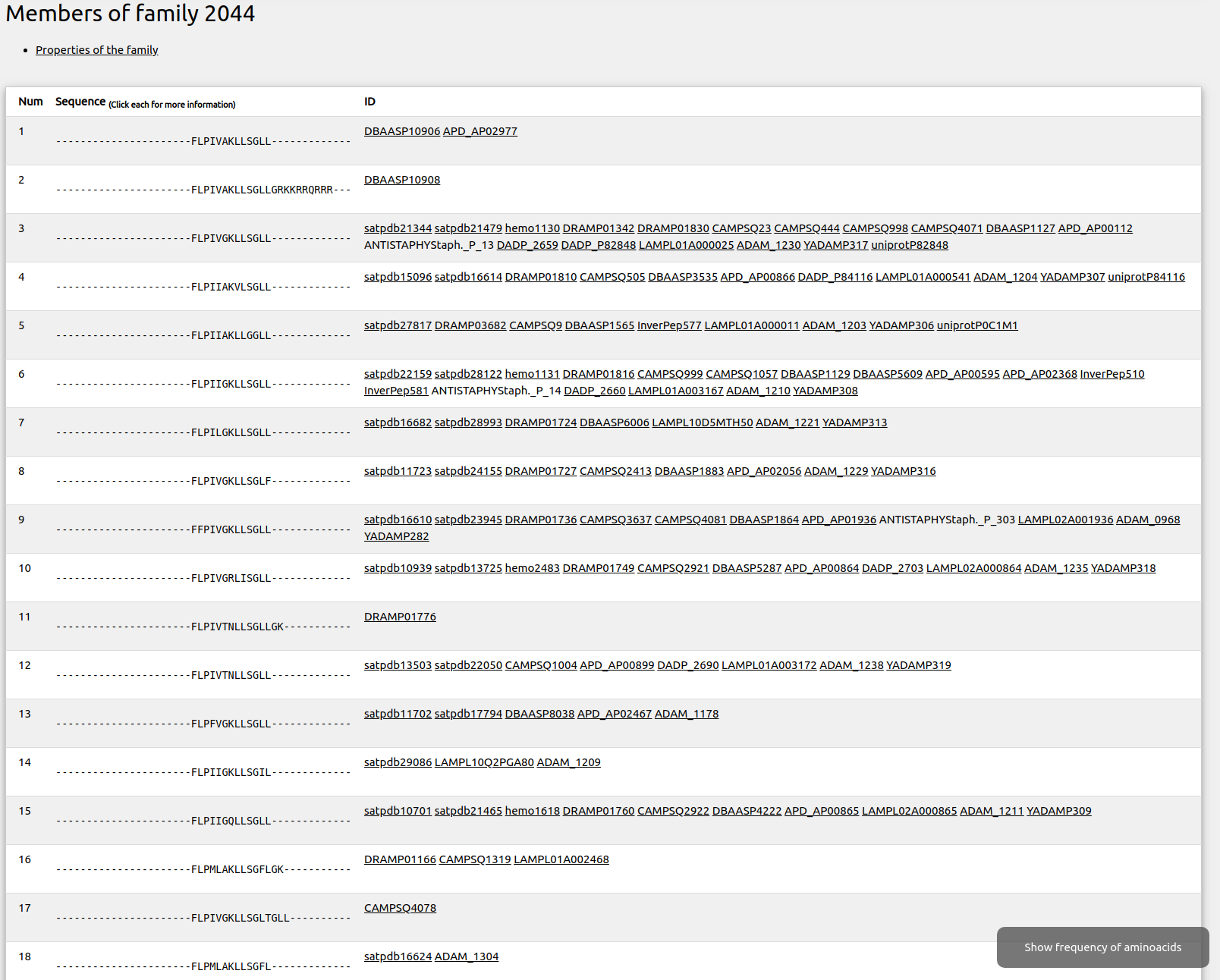
***Screenshot 4.***

# ADAPTABLE TUTORIAL - CASE EXAMPLE n.1

## Designing new peptides active towards a specific organism and highlighting motifs

### The case of *Shigella*

Suppose you want to design a new antimicrobial peptide active against *Shigella* sp. You want to be inspired by the most active entries in the database, so you look for peptides which are active at concentrations lower than 1 μM. Go to the “FAMILY GENERATOR” section on the left bar. For the Calculation label, you can choose a name such as “peptides_against_shigella”. As you do not want to restrict the generation of families only taking into account “Aurein” peptides (provided as an example), remove all from “Peptide name” field. Go to “Target Organism” and write the name of the organism (or part of it) and the maximum μM activity value just like in *screenshot 1.1.* You will also need to choose the username (“user” in the example) to be able to access your experiments:

***Screenshot 1.1.***


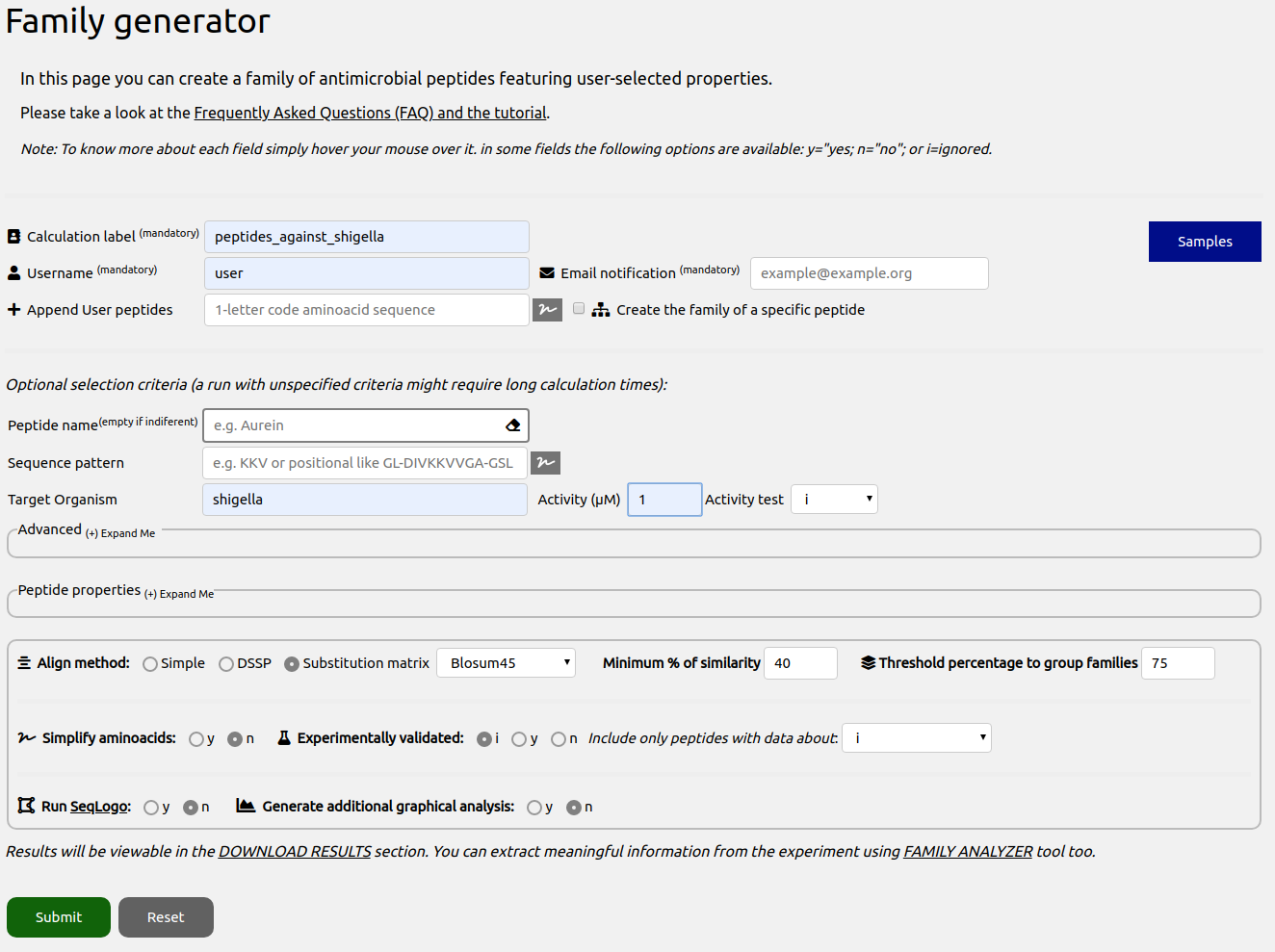


You might want to see a detailed analysis so you can also set the options “Generate additional graphical analysis” and “Run SeqLogo” at the end of the page to yes (“y”).

Click on “Submit” at the end of the page. After the calculation is finished, a page summarizing the properties of each generated family will be shown (always accessible from “FAMILY ANALYZER” section). You can also go to the “DOWNLOAD RESULTS” section on the left bar to download all the generated files. In both case you will be asked to type your username:

***Screenshot 1.2.***


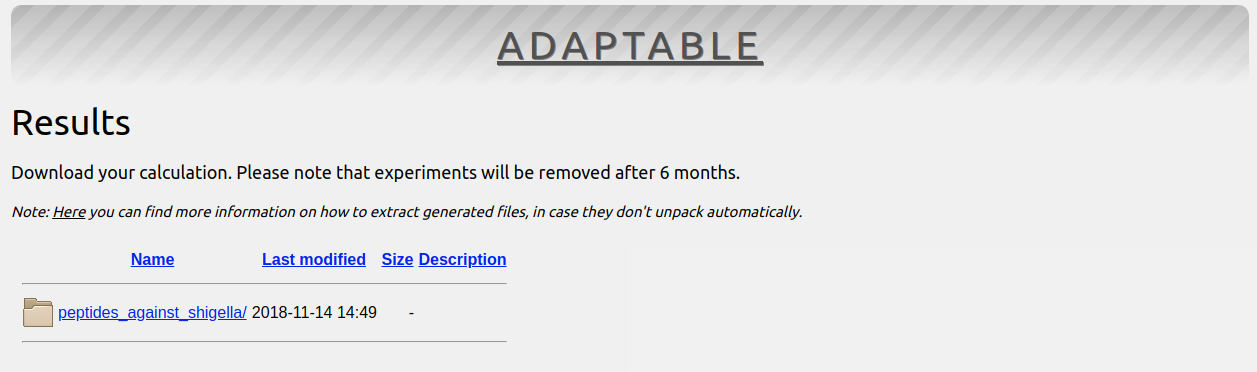


Open the directory of your calculation “peptides_against_shigella”. Here is what you see:

***Screenshot 1.3.***


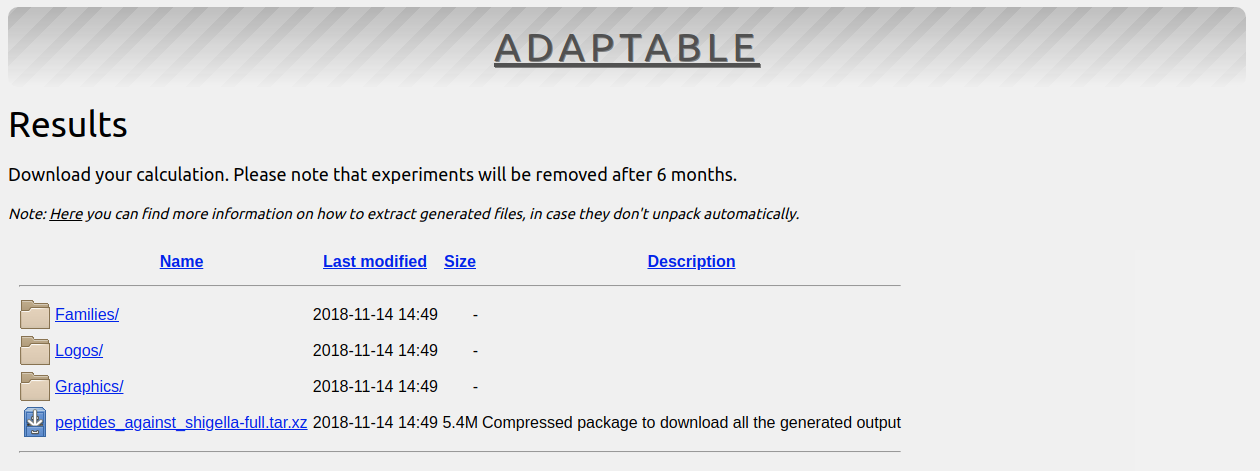


You will see a compressed package to download all the generated files, additionally, in the subfolder “Families” you will find the full information for each family (as a whole html file showing different properties and statistics), in the subfolder “Logos” you will find the logos generated by Weblogo and in “Graphics” you will see the generated additional graphical analysis files.

We will go to “FAMILY ANALYZER” section, that includes tools that will allow to parse the generated information in a more detailed way. It will ask for the username and, after that, the following section will be shown to allow choosing the desired user experiment:

***Screenshot 1.4.***

***
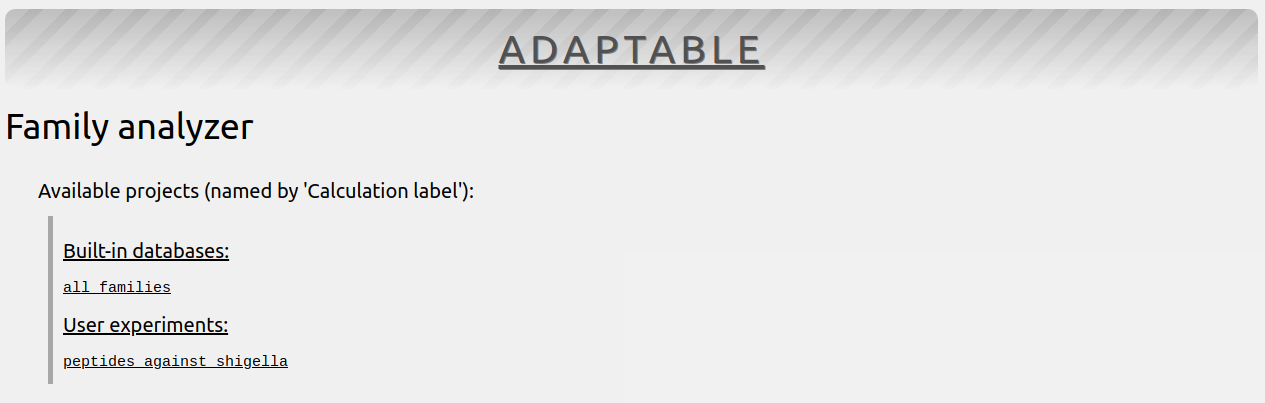
***

After going into our experiment, we can see a page that will provide us with some information (Screenshot 1.5).

The run has generated a total of 8 families with more than one member (you can see the rest clicking on “Expand Me to show families with 1 member”. Regarding the global properties of the families, you can review a summary and the full html output for each.

It is also shown that this run has generated Logos and Graphical plots that can be accessed directly from here too.

Finally, it’s shown that 2 of the 21 generated families are similar among them for the case the user wants to take this into account when reviewing the features of each family.

Going into the “Detailed analysis of family members” we will be able to select the families to analyze for getting more detailed information.

***Screenshot 1.5.***


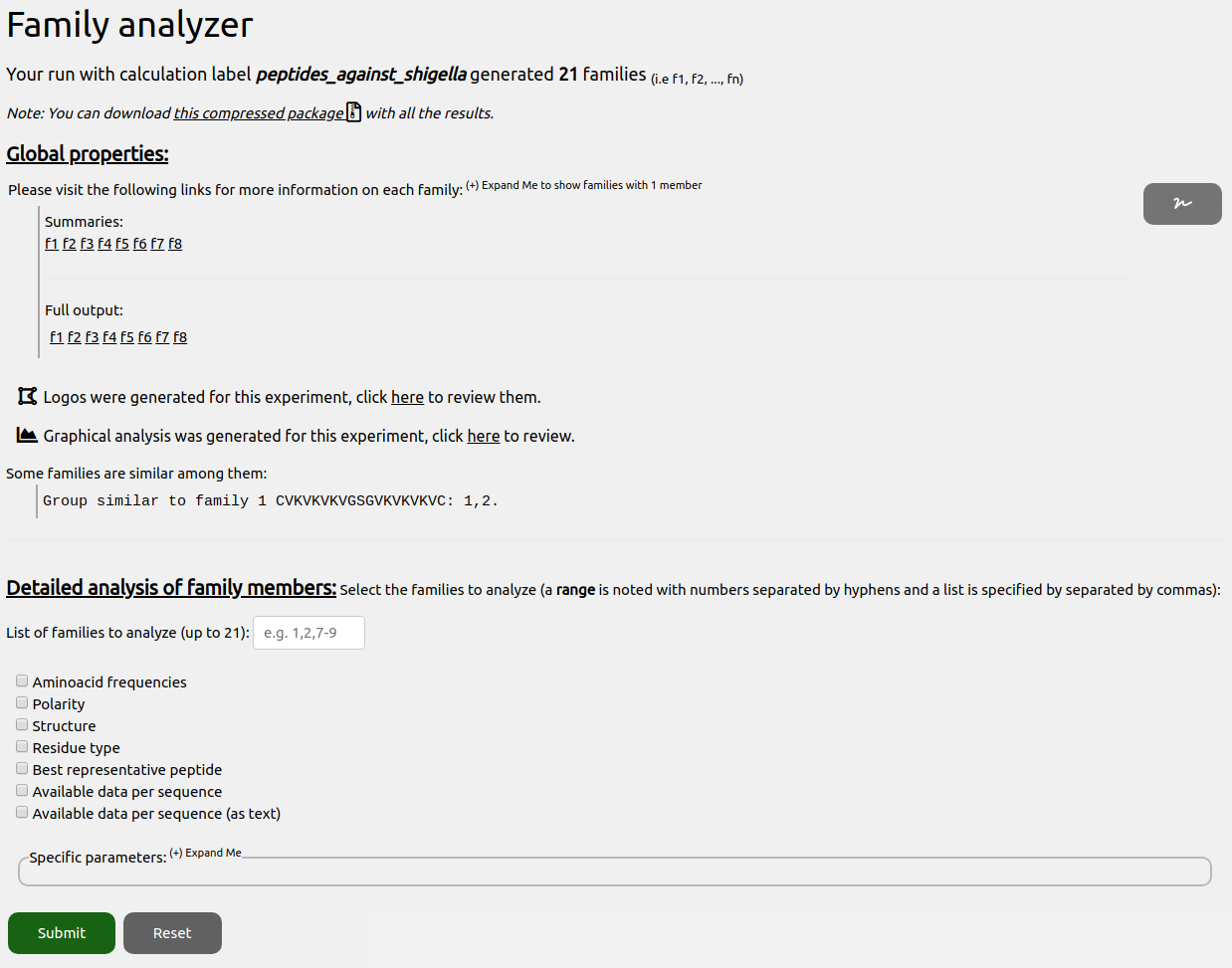


Based on sequence alignment, ADAPTABLE has classified all peptides of its database in groups of sequence-related peptides (families), each represented by a single sequence chosen among the members of the family (called the father). The father has the highest resemblance to all other peptides. In this terminology, each family is composed by a father and multiple sons (the other members of the family). This classification is purely based on sequence; it is therefore independent on the activity or other properties.

We will choose to review the members of family 1 and get them shown according to the “Aminoacid frequencies”, “Polarity” and type of residue.

Among the 7 members, the first sequence is the one which has been used as reference to build the family, and is therefore called the “father”.

Judging by the frequency of occurrence, it seems that some amino acids (in red) are highly repeated in the same position and therefore potentially important for the antimicrobial mechanism of action. The statistical analysis performed at the end of the calculation will help us to better understand this information (see below).

Coloring the sequence alignment by residue type or polarity can sometime reveal other features (as the presence of amphipathic helix).

***Screenshot 1.6.***

***
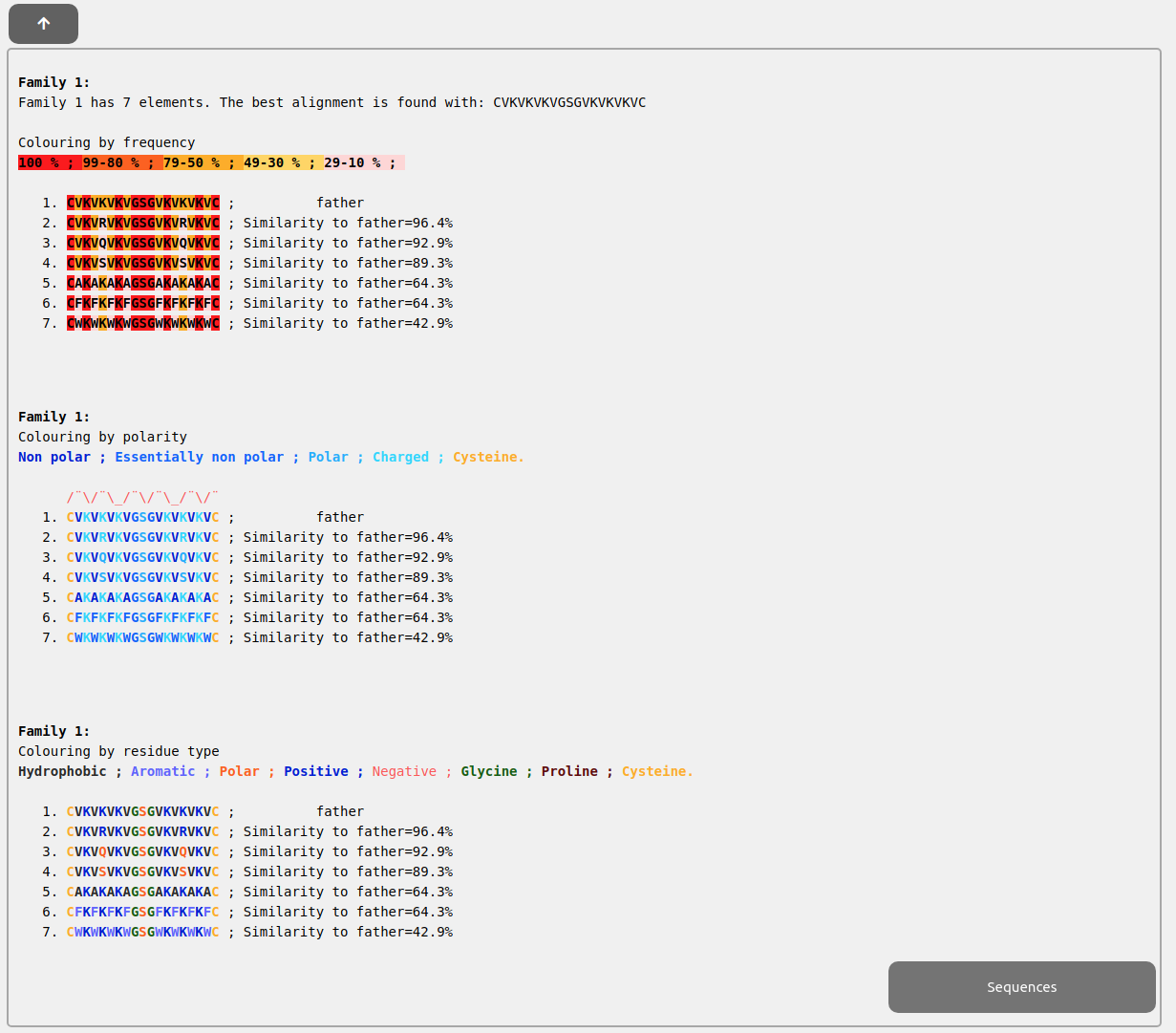
***

In this case, the interpretation is not straightforward. Polar and nonpolar residues do not seem to alternate with the frequency of a helix (3.5 residues per turn). The secondary structure prediction, along with the DSSP structure (“Structure” option) confirms that a helical structure, commonly found in antimicrobial peptides, is not expected in this case:

***Screenshot 1.7.***


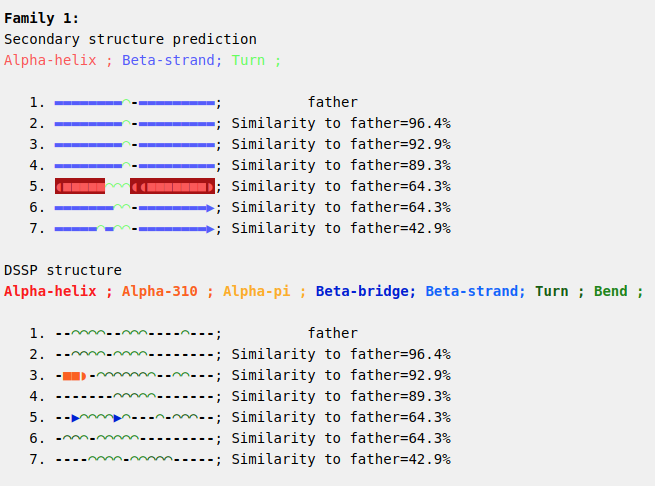


We can also see a summary of all the information available for the family clicking on “Available data per sequence”:

***Screenshot 1.8.***


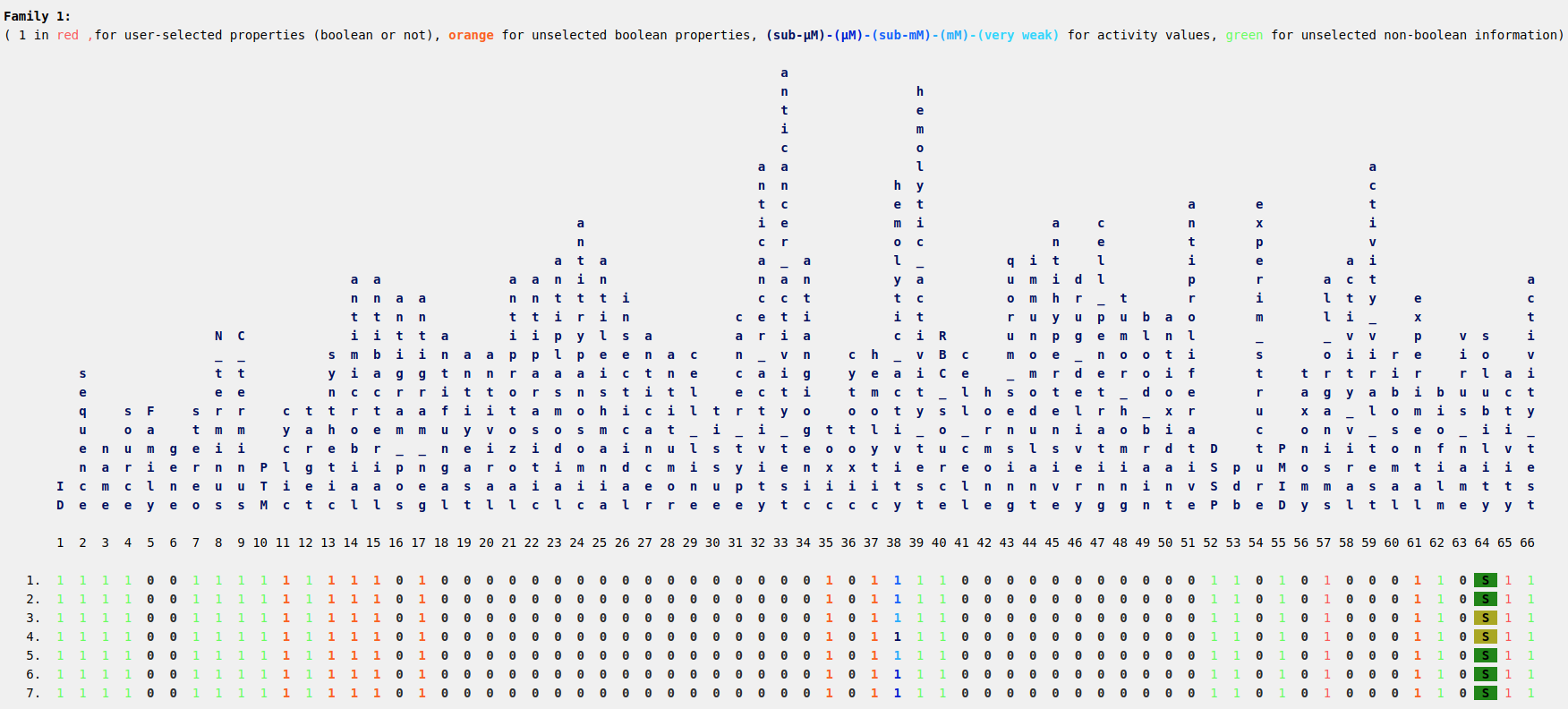


In this table, each line corresponds to one member of the family and each column corresponds to a property (“1” indicates presence of the property).

But a more readable output showing several statistics and tables can be obtained in the generated HTMLs with the full output as accessed in “Global properties” section:

***Screenshot 1.8.***


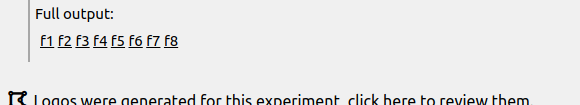


In this case, we will click into f1 output and we will see the whole output divided in multiple sections that can be navigated using its left bar to jump from one section to other.

***Screenshot 1.9.***


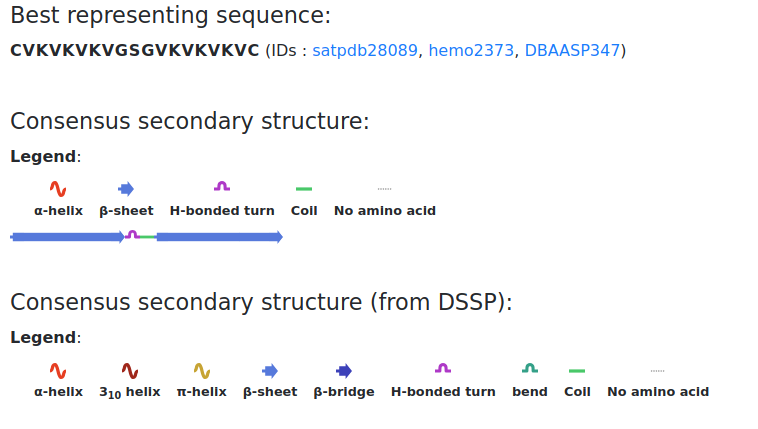

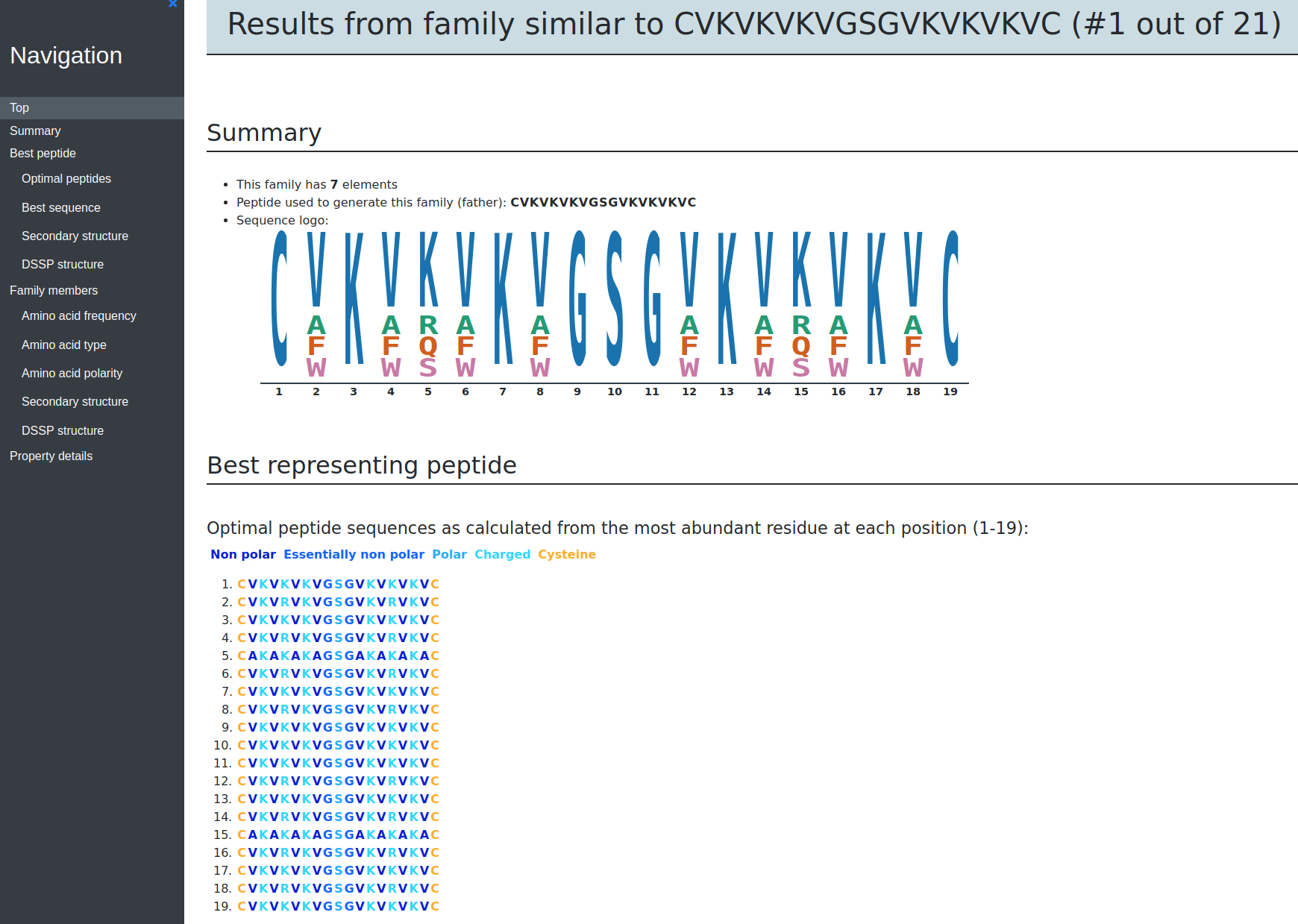


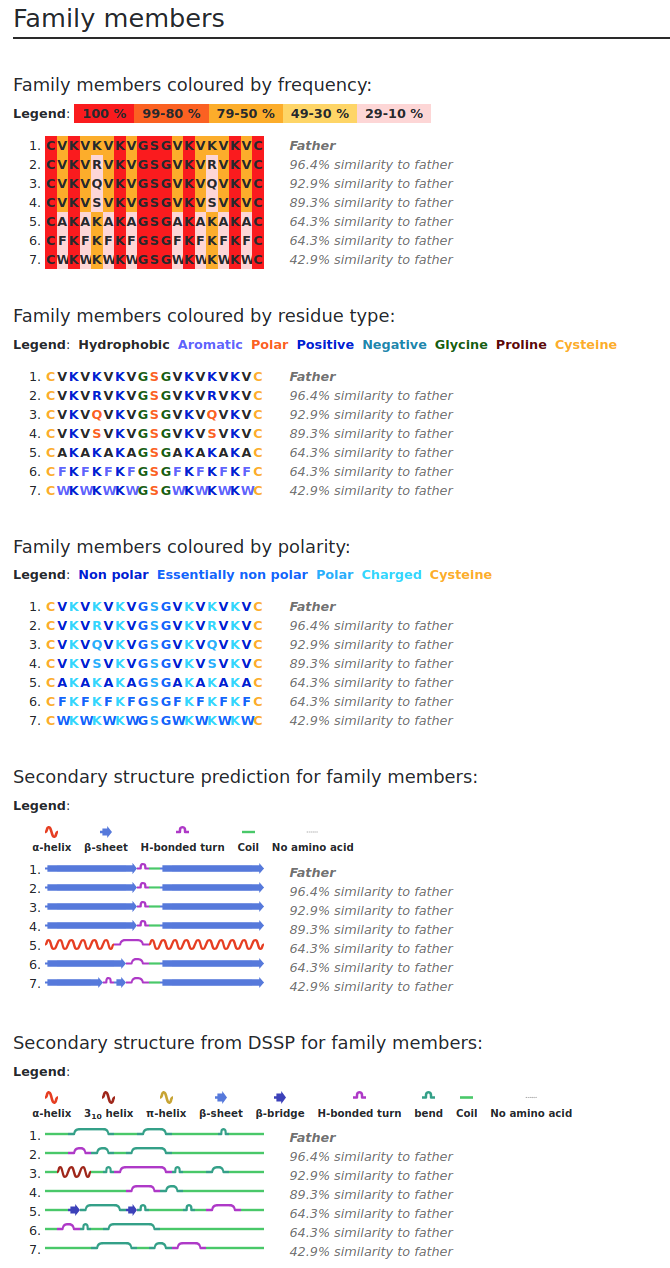


A table is also plotted here showing a summary of all the relevant properties:

***Screenshot 1.10.***


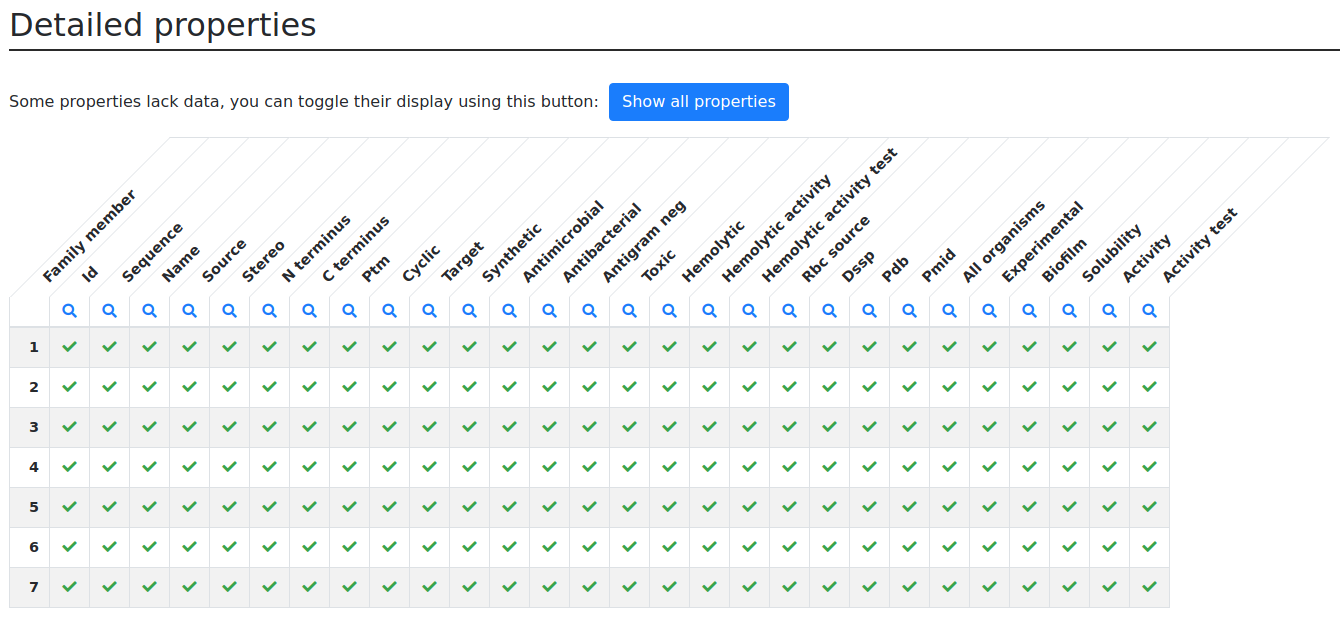


We immediately see that these peptides are mostly cyclic, synthetic, actives against gram-negative organisms. We also see that this family tends to be hemolytic and toxic (column 38). Therefore, chemical modifications should be performed to reduce their toxicity.

After that table, some more details are listed:

***
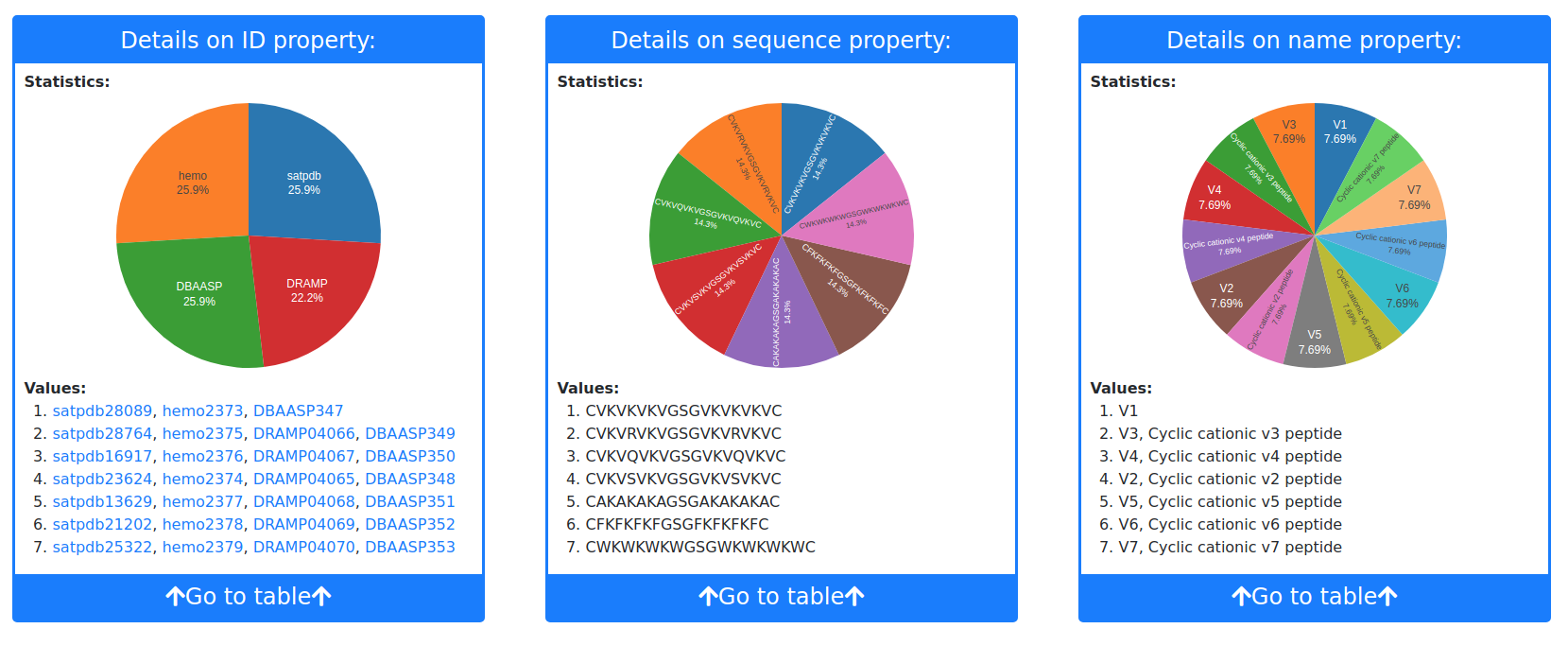
Screenshot 1.11.***

An additional graphical statistical analysis on the amino acid composition and location helps clarifying the properties of this family. The analysis can be found in the subdirectory Graphics in the “DOWNLOAD RESULTS” section on the left bar or simply through the link in “Global properties” section of “Family Analyzer”.

***Screenshot 1.12.***


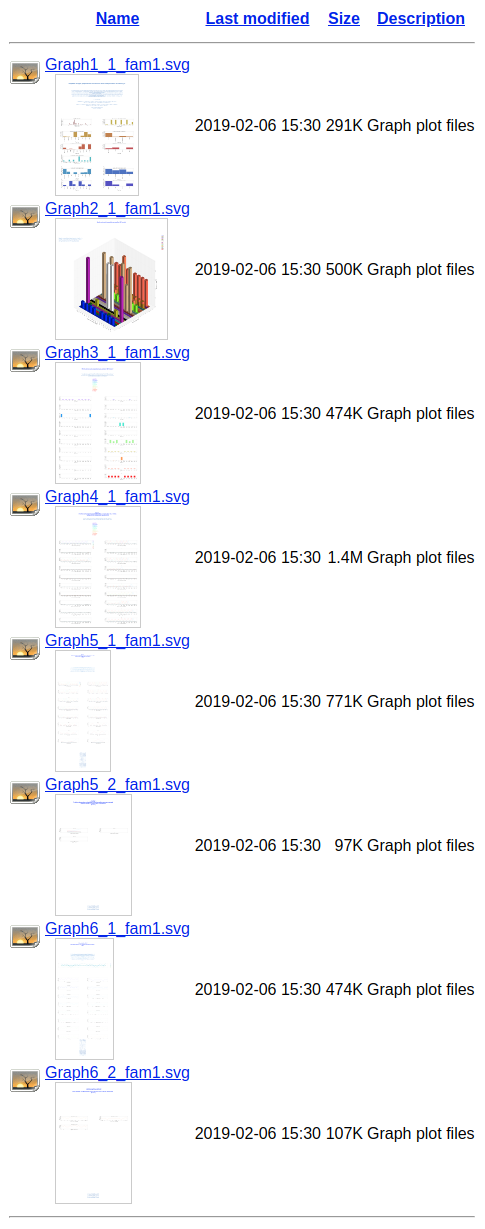


We find 6 different types of graphs. In graph 1, we can see that the members of family 1 contain on average 5 lysine residues (K) and 4.5 valines (V) but also 2 cysteines (C) per peptide. Hydrophobic and positively charged amino acids dominate the sequence and amino acid side chains tend to be of reduced size.

***Screenshot 1.13.***

***
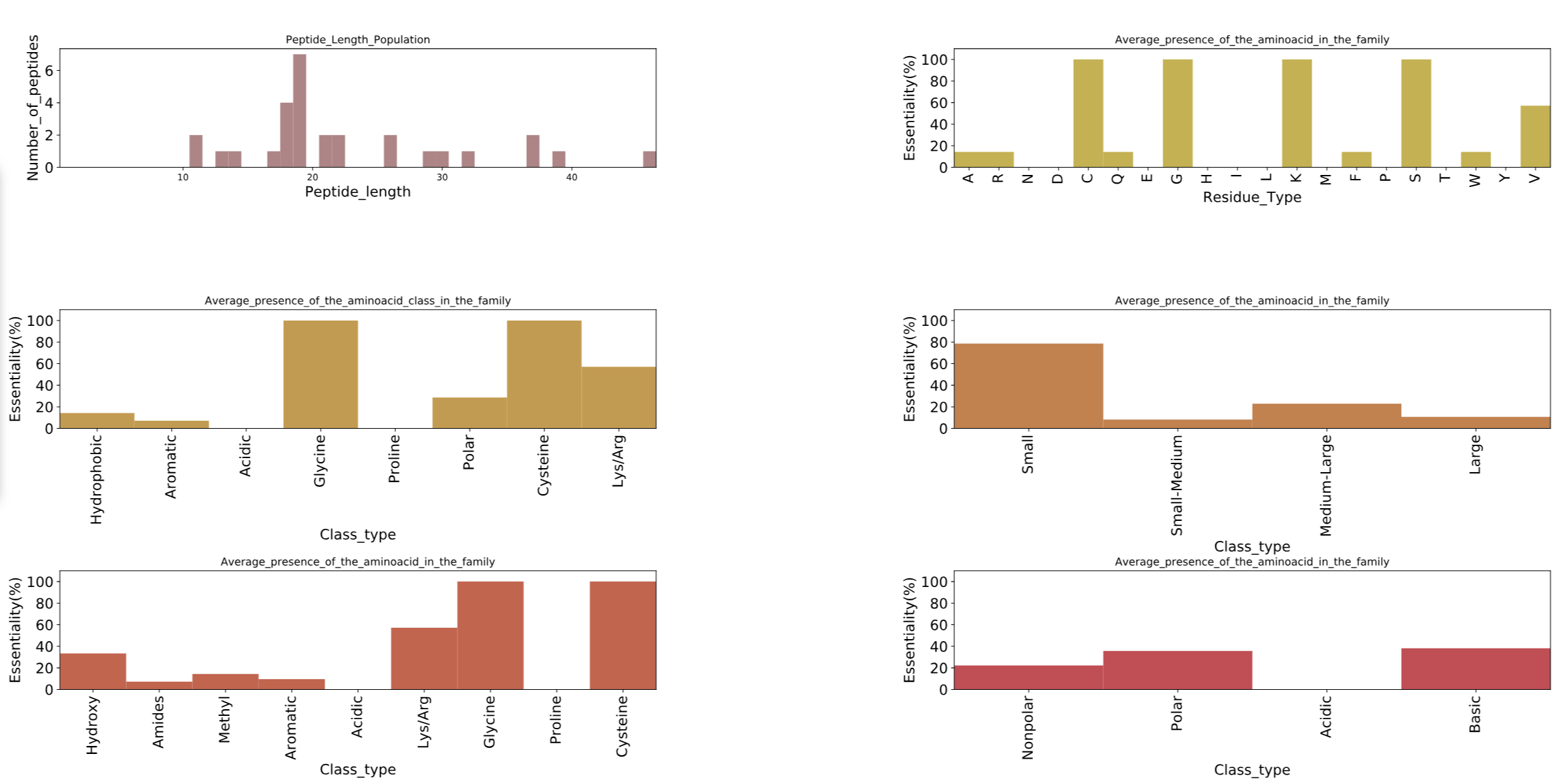
***


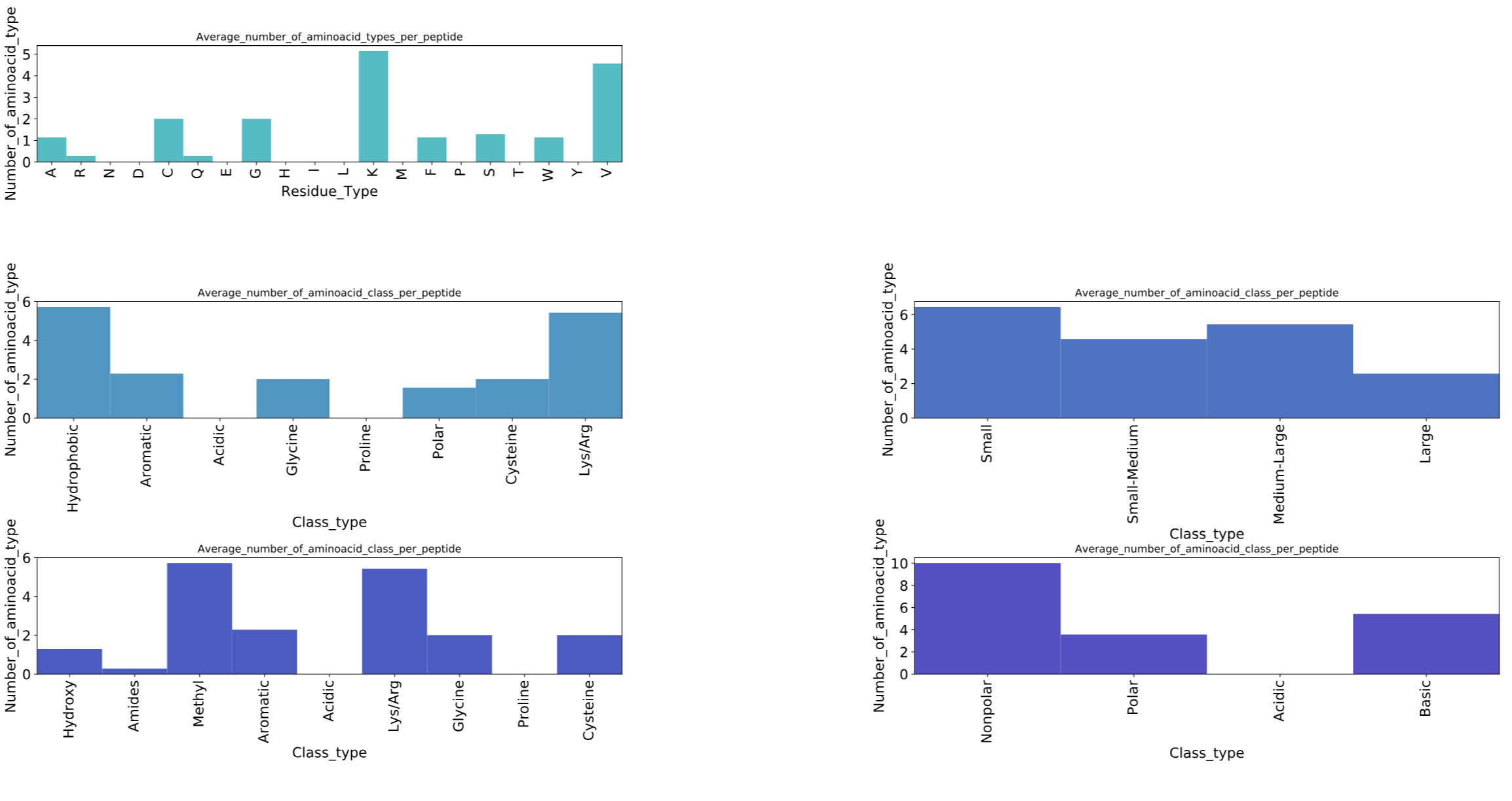


In graph 2, a 3D scheme (its 2D version is in graph 3) shows that the position of valines (green) and of lysines (cyan) along the sequence does not seem to matter. On the contrary, Cysteine (magenta) residues tend to be at the termini. This might suggest the presence of a head to tail disulphide bond (in the “data table” previously examined the peptides are actually reported to be cyclic).

***Screenshot 1.14.***


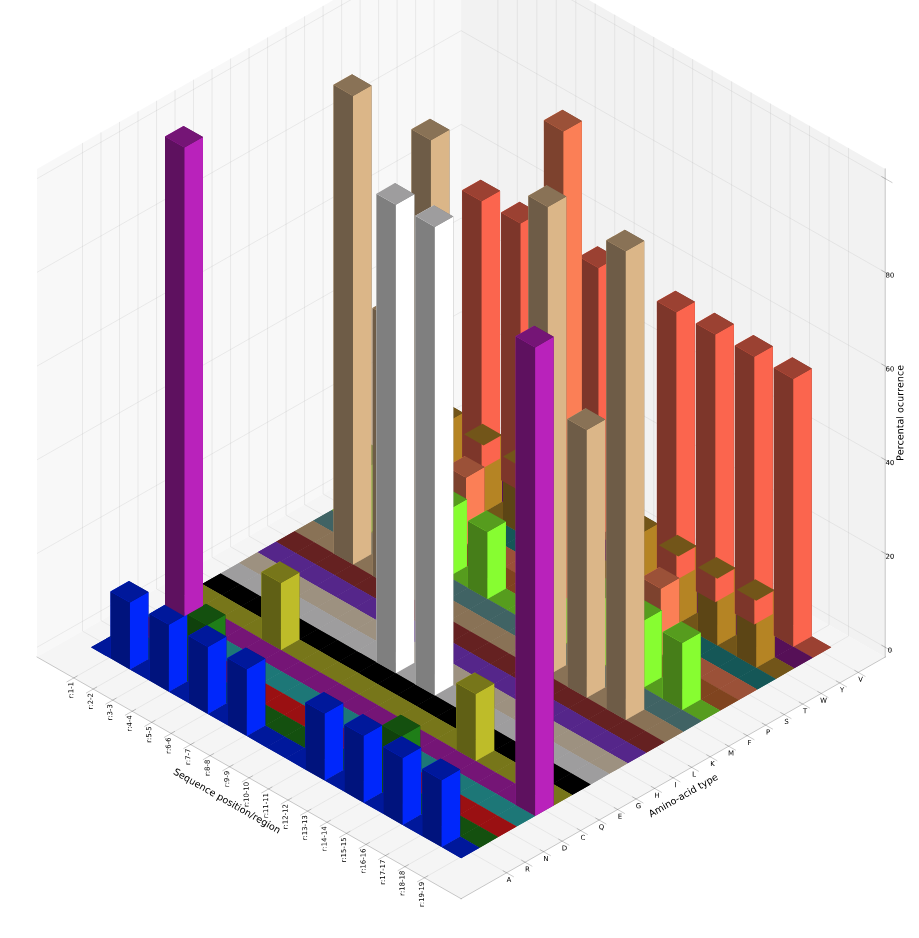

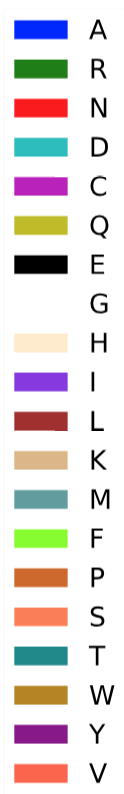


To confirm this hypothesis, we can check the probability to find a cysteine in last position when a Cysteine is present in the first position. This information can be retrieved from graph5 of family 1:

***Screenshot 1.15.***


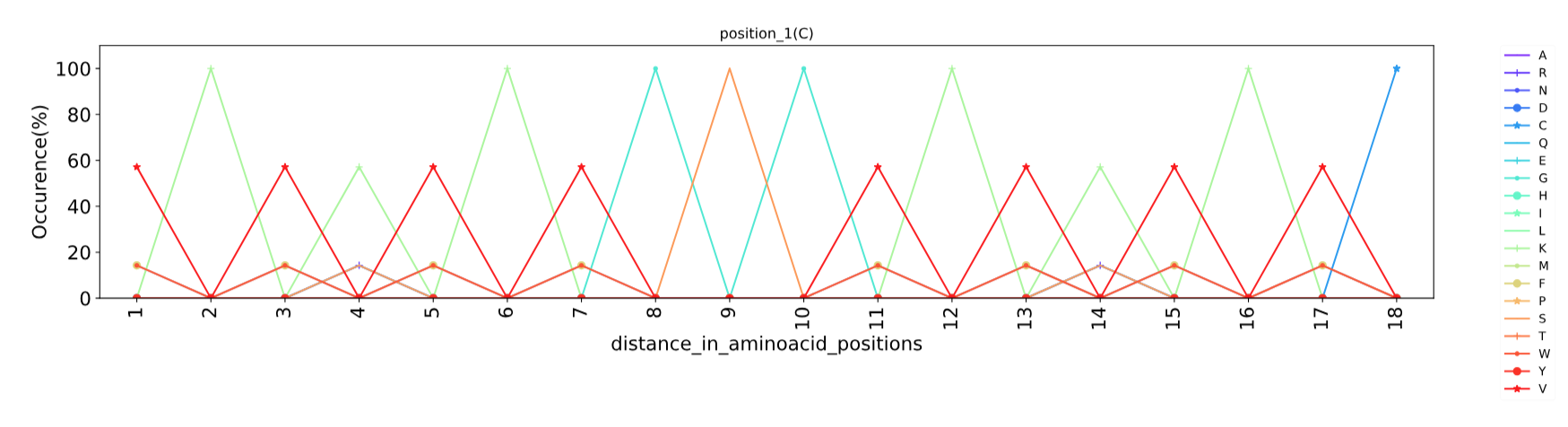


In this graph, different amino acid types have different color code.

When a Cysteine is present in position 1 the probability to find another Cysteine (blue line) 18 amino acids (aa) apart, is 100%. This might sound as trivial information in the present case (these are synthetic peptides) but it might be very useful for peptides whose structure have not been studied yet.

Graph 4 analyzes motifs (groups of amino acids in well-defined relative positions within the sequence), an important information often shedding light on the mechanism of action. Motifs are found by calculating the position-independent probability to find a residue type at a certain distance from a reference residue type (e.g. if you have an Alanine in a peptide of the family, you can calculate which is the probability to find each of the 20 amino acid types n positions apart).

In our case, when C is present, graph 4 states that we have a 100 % probability to find K (green) 2, 6, 12 and 16 positions apart, G (cyan) 8, 10 position apart, S 9 position apart and C 18 positions apart. This means that C-K---K-GSG-K---K-C is a common motif for family 1.

***Screenshot 1.16.***


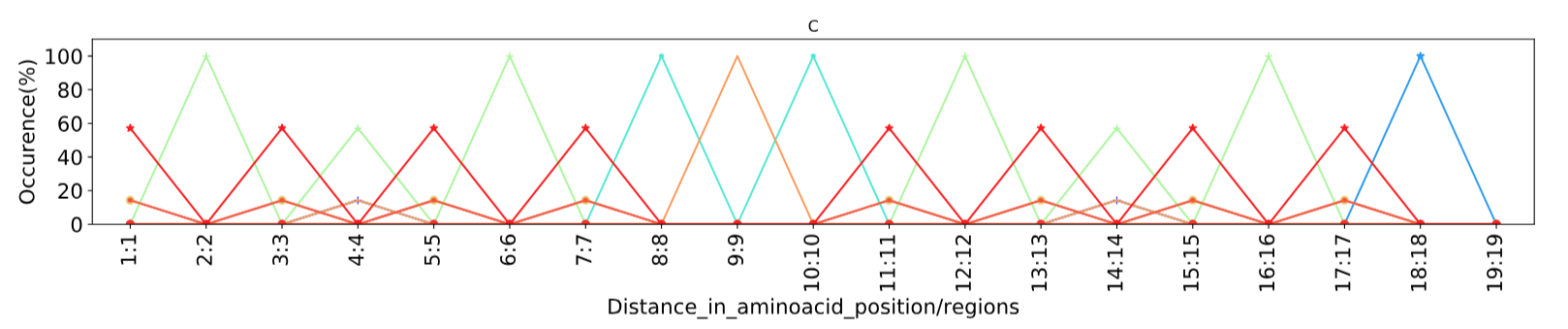


Among this family of peptides highly active against *Shigella* sp., we have highlighted the “father”, which is the peptide which better represents the sequences of all other peptides of the family. Can we design a new sequence better resuming the properties of family 1, including the father?

Creating a peptide by concatenating the most frequent residue per position is a tempting but inefficient way to solve this problem. The resulting peptide would be a chimera where the synergic action of specific amino acid types in optimal relative positions would be likely lost. However, starting from the most abundant amino acid in each position, we can fill all other positions with the most frequent amino acid found in related peptides. As shown by the logo image, the most abundant amino acid in position 8 is Valine (you can find the logo image in the logos directory of “DOWNLOAD RESULTS” section). This feature of ADAPTABLE is powered by WebLogo/SeqLogo software; <http://weblogo.threeplusone.com/> , Department of Plant and Microbial Biology, University of California, Berkeley):

***Screenshot 1.17.***


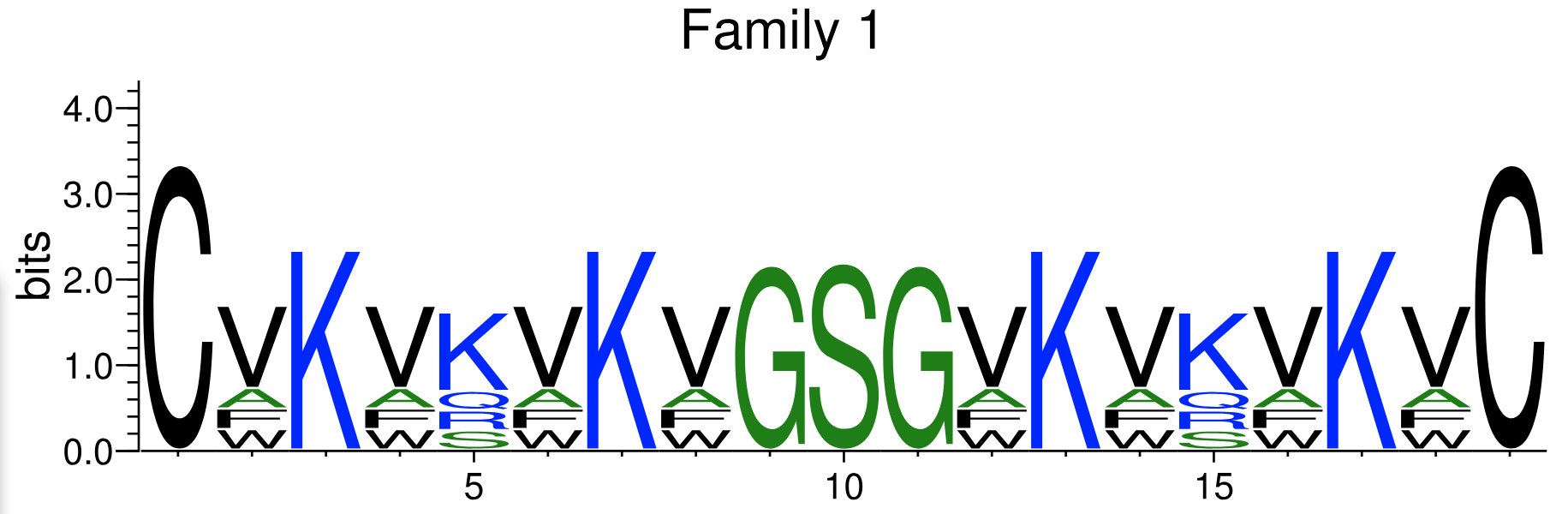


Analyzing all peptides with Valine in position 8 (see graph 6), we can find that the most frequent amino acid in position 1 is C, in position 2 is V and so on. As shown in the figure, we can do the same starting from position 7 or 9 or 10.

***Screenshot 1.18.***


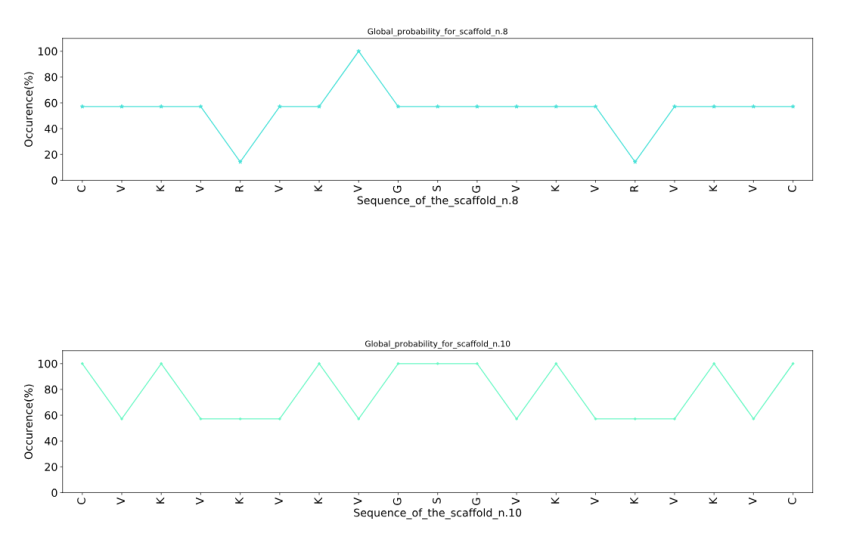

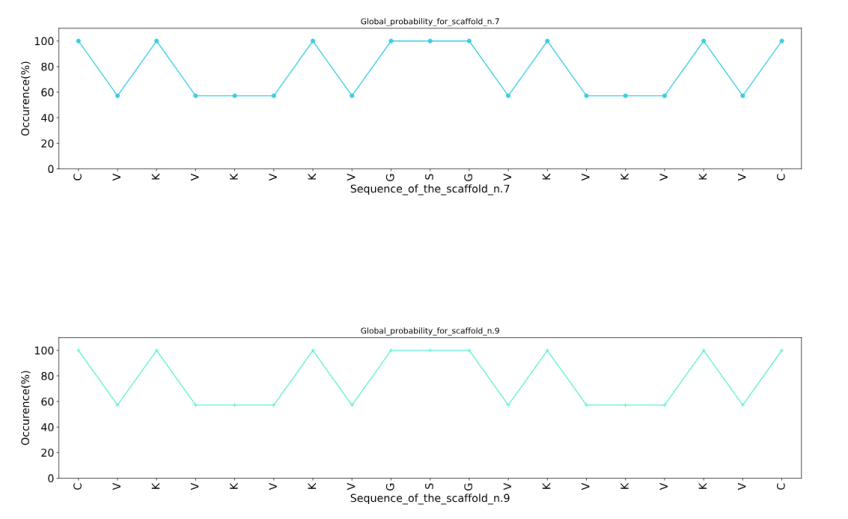


In this way, we can create many different optimal peptide sequences, one per each position. The best representative peptide is chosen selecting the curve maximizing the sum of probabilities over all positions (the area of the graphs).

The optimal peptide can be found searching for “Best representative peptide”.

***Screenshot 1.19.***


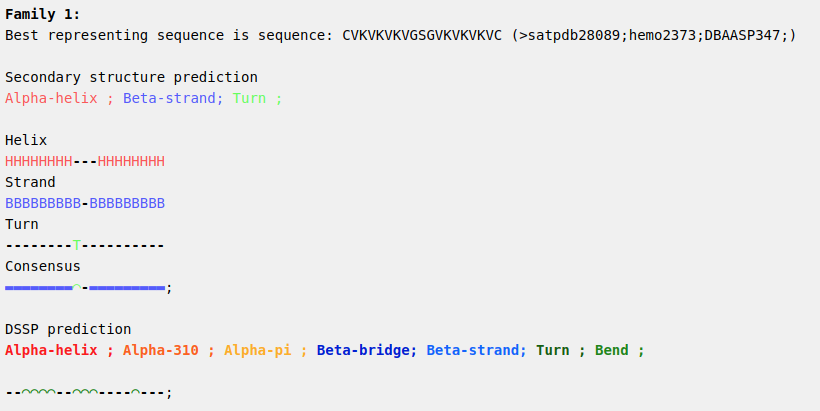


You can also specify more parameters simply clicking on the box to expand it and show them.

***
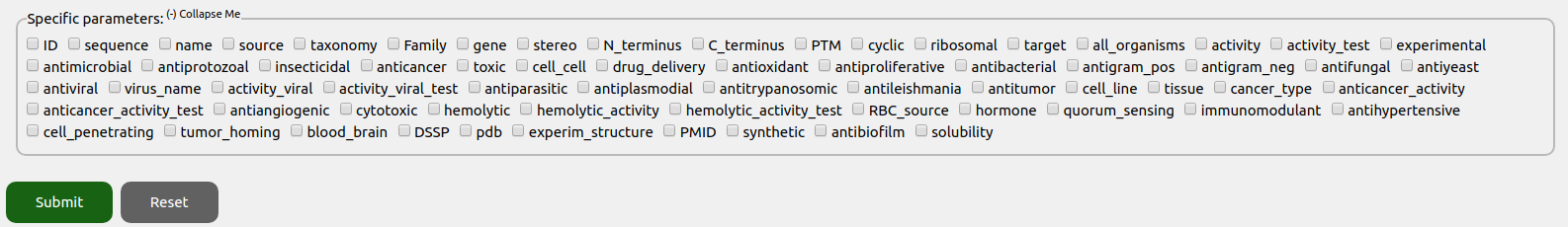
Screenshot 1.20.***

For example, selecting “Aminoacid frequencies”, “Best representing peptide” and “PMID” (the reference to the publication on the selected peptides) you can visualize all this information in one page:

***Screenshot 1.21.***

***
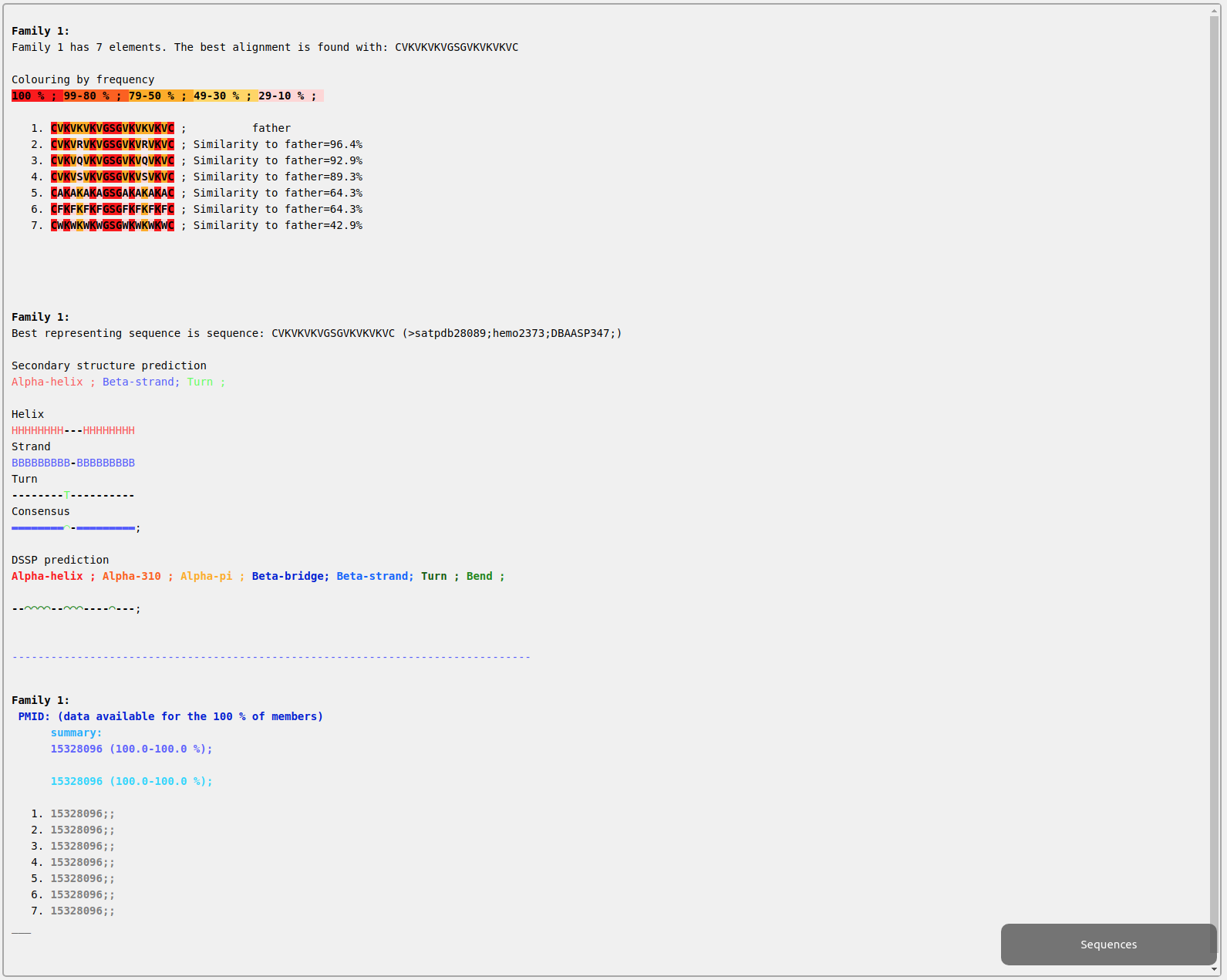
***

Using the PMID 15328096 we can check the publication related to the family of peptides and have a confirmation of what we have “discovered”. You can also visit the page for getting the details of each family. The template peptide for this family of synthesized peptides, which was not present in the database, coincides with our optimal sequence. The family do has a disulphide bridge connecting the N and C terminus. No helical structure is present.


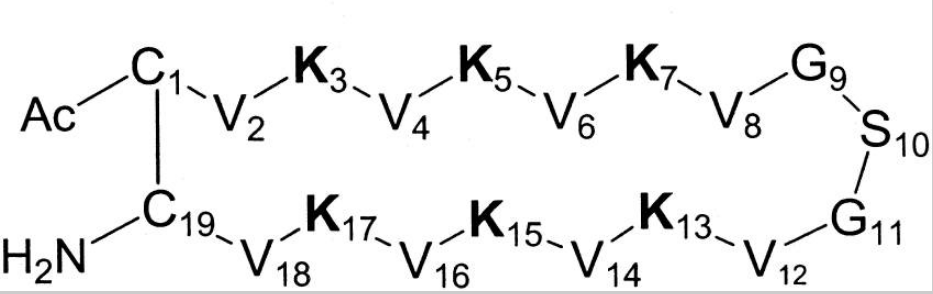


This image was taken from Frecer, V., Ho, B. & Ding, J. L. De novo design of potent antimicrobial peptides. Antimicrob. Agents Chemother. 48, 3349–3357 (2004).

# ADAPTABLE TUTORIAL - CASE EXAMPLE n.2

## Predicting the antimicrobial or anticancer activity of a generic peptide sequence

### The case of random new peptides

Suppose to have rationally designed an antimicrobial or anticancer peptide targeting microbial or cancerous cell membranes. Your sequence is entirely new and you want to predict its activity based on experimental data on similar peptides. Just for the sake of demonstrating the potentiality of ADAPTABLE in testing new sequences, we will use a completely random sequence: LLKKPPRWQTRGHKWCQRTP .

In order to predict the activity we will tell ADAPTABLE to “Create the family of a specific peptide” using this one as reference. We will give the name “test_LLKKPPRWQTRGHKWCQRTP” to calculation.

***Screenshot 2.1.***


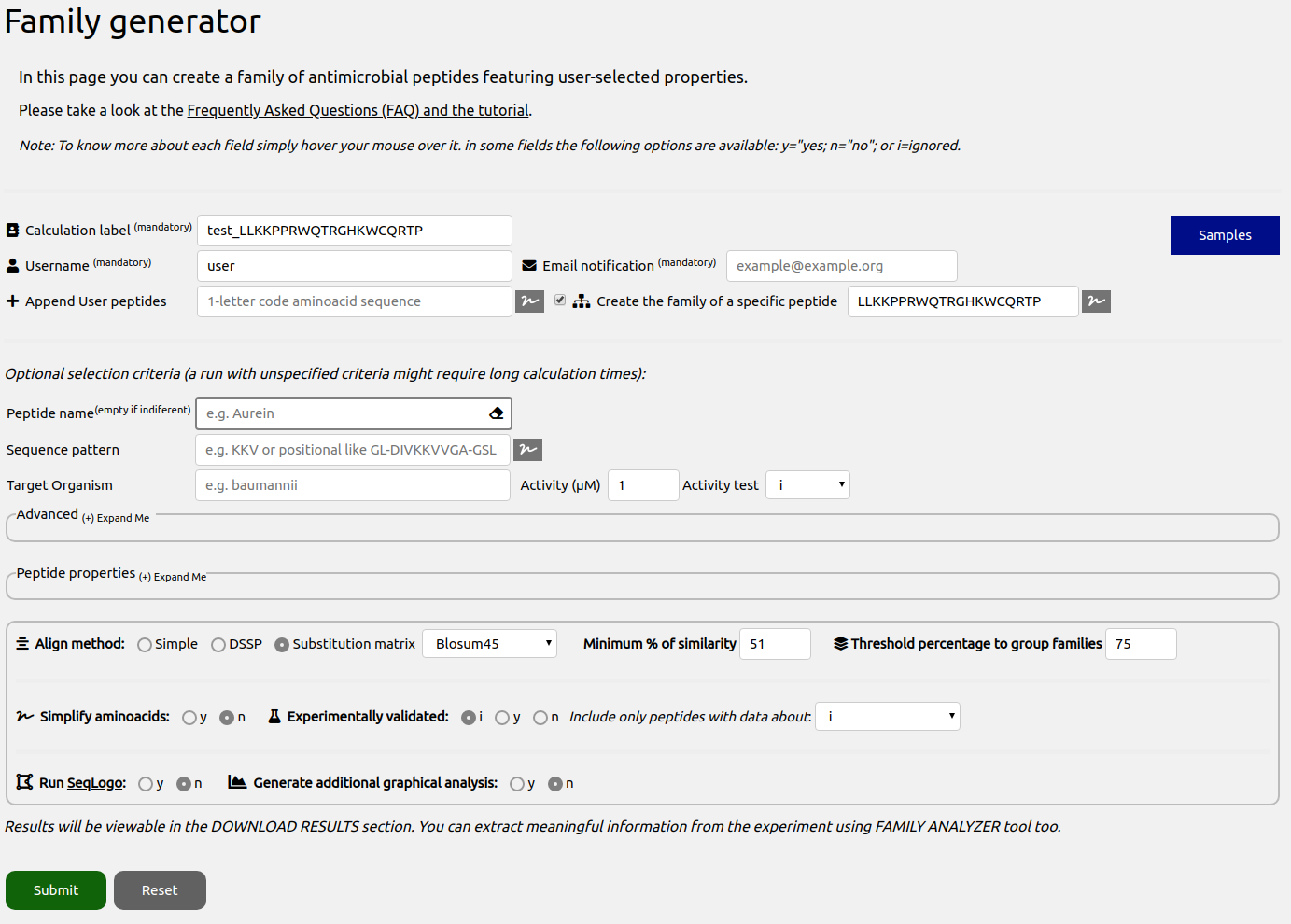


This tool allows to compare a test sequence (in our case LLKKPPRWQTRGHKWCQRTP), with the full database. In our case, running the prediction did not produce any interesting result. Despite the fact that our sequence contains many amino acids frequently found in antimicrobial peptides (K, A, V, R), the sequence did not resemble any of the peptides of our database. This, of course, does not mean that our random peptide does not exhibit antimicrobial or anticancer properties. However, if active, we can estimate that its mechanism of action is not common in nature.

In order to get more insight in the possible activity of our test peptide, the calculation can be repeated “in simplified space” (setting to “simple” the align method), where residue types are compared by their properties. In this way, hydrophobic residues (A, V, I, L, M) are represented by A, negative residues (D, E) by D, positive residues (K, R) by K, aromatic residues (W, Y, H, F) by F, polar residues (S, T, N, Q) by S, modified amino acids by (Ⓜ). Gly, Pro, Cys are treated individually.

***Screenshot 2.2.***


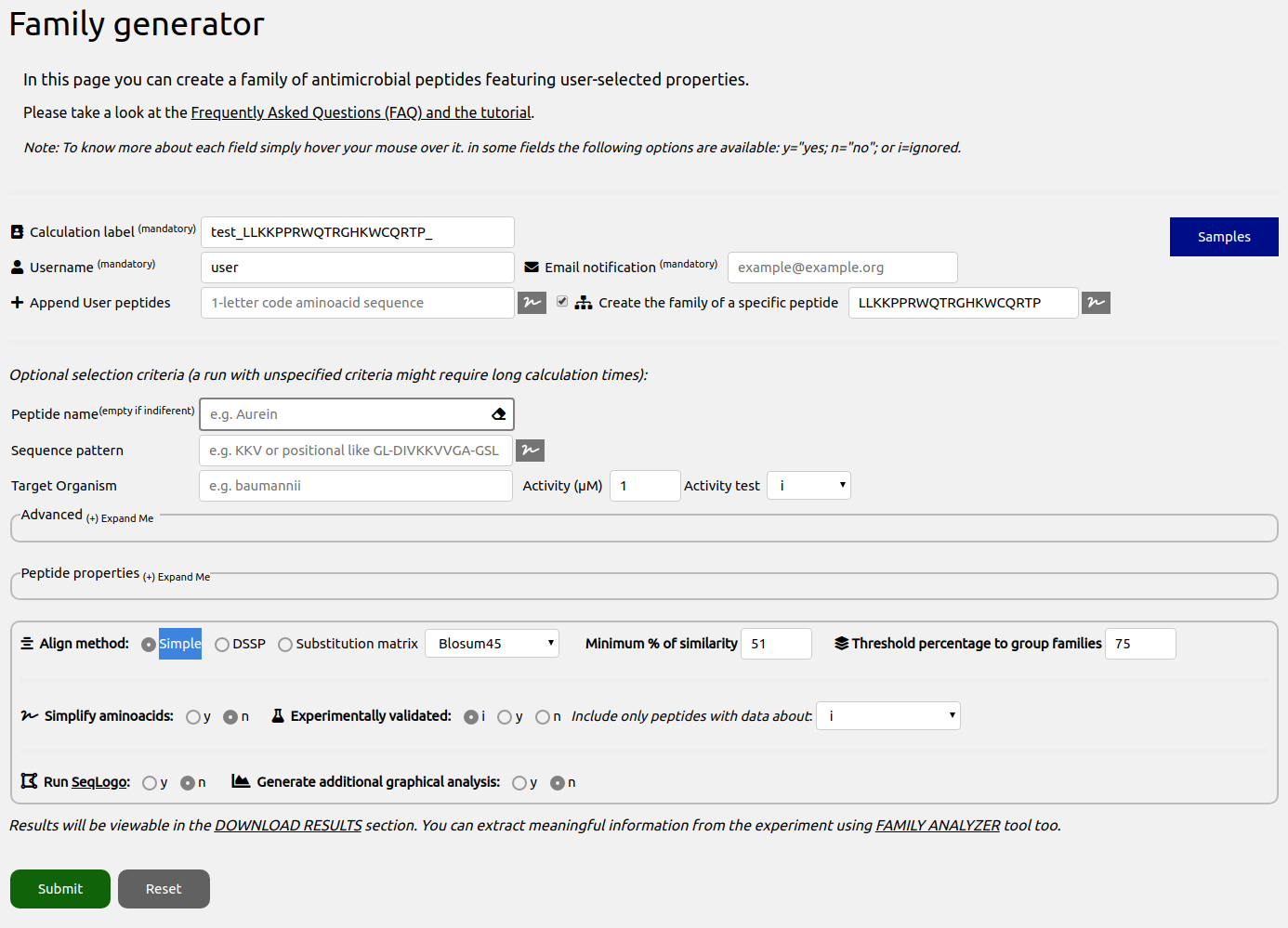


After the calculation, a page summarizing the features of the generated family will be shown. We can analyze the results more deeply by going to the “FAMILY ANALYZER” page and typing our user we can choose our run test_LLKKPPRWQTRGHKWCQRTP_:

***Screenshot 2.3.***

***
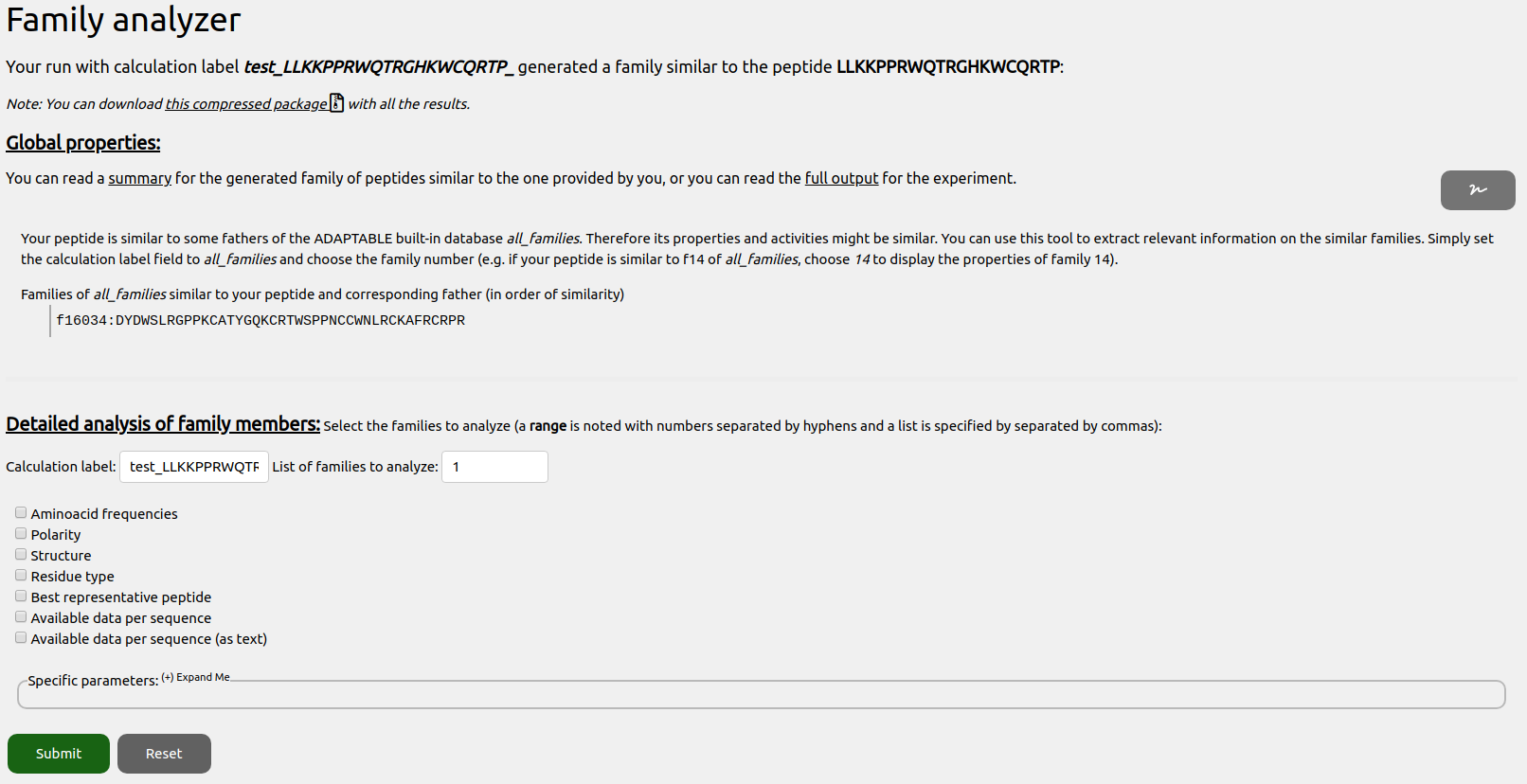
***

Our test peptide was able to generate a family of 3 similar peptides (the family can be visualized by clicking on the “Submit” button in the lower part of the page with the option “Aminoacid frequencies” checked). Remember that when running the experiment you can tune the minimum similarity to get peptides included in the family, and change the alignment matrix and method:

***Screenshot 2.4.***


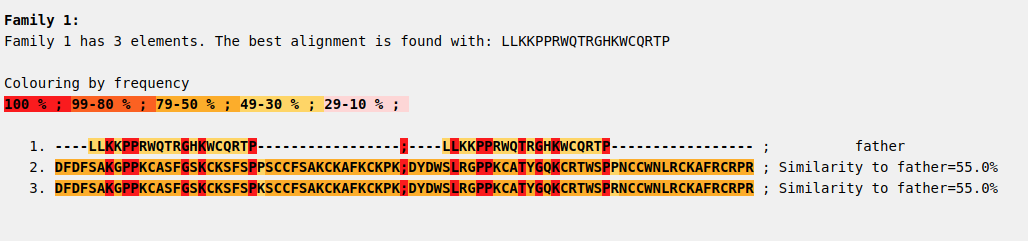


In the family, the first sequence is our test peptide, followed by similar database sequences with decreasing similarity. We can now click on the “Up arrow” button in the upper part and re-run the analyzing tool to retrieve other information.

In the *Screenshot 2.3*, ADAPTABLE also reports that the test peptide is similar to the father of the family 16064 from “all_families” experiment (a built-in experiment that aims to group all the peptides into families with similar sequence). Therefore, it would be interesting to see its properties. In order to do so, we can use “all_families” as calculation label and we can type “16034” in the “List of families to analyze” field, as in *Screenshot 2.5*.

***Screenshot 2.5.***


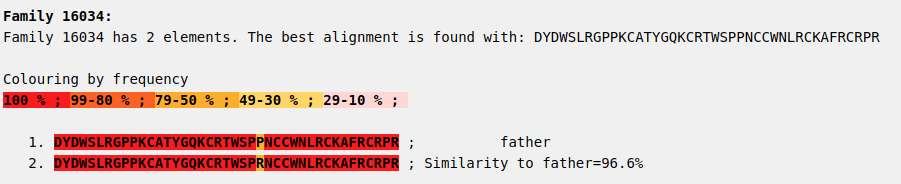


Selecting “Available data per sequence” and clicking on “Submit”, we can see that the similar family 16034 is composed of members with antibacterial activity.

To conclude, out test peptide might have antibacterial activity although its sequence is not strikingly similar to any entry of the database.

Let’s now try with a different peptide: GFGMAAKLAKK.

In this case, going to the “FAMILY ANALYZER” section and looking for “Aminoacid frequencies” information, we observe something more interesting:

***Screenshot 2.8.***


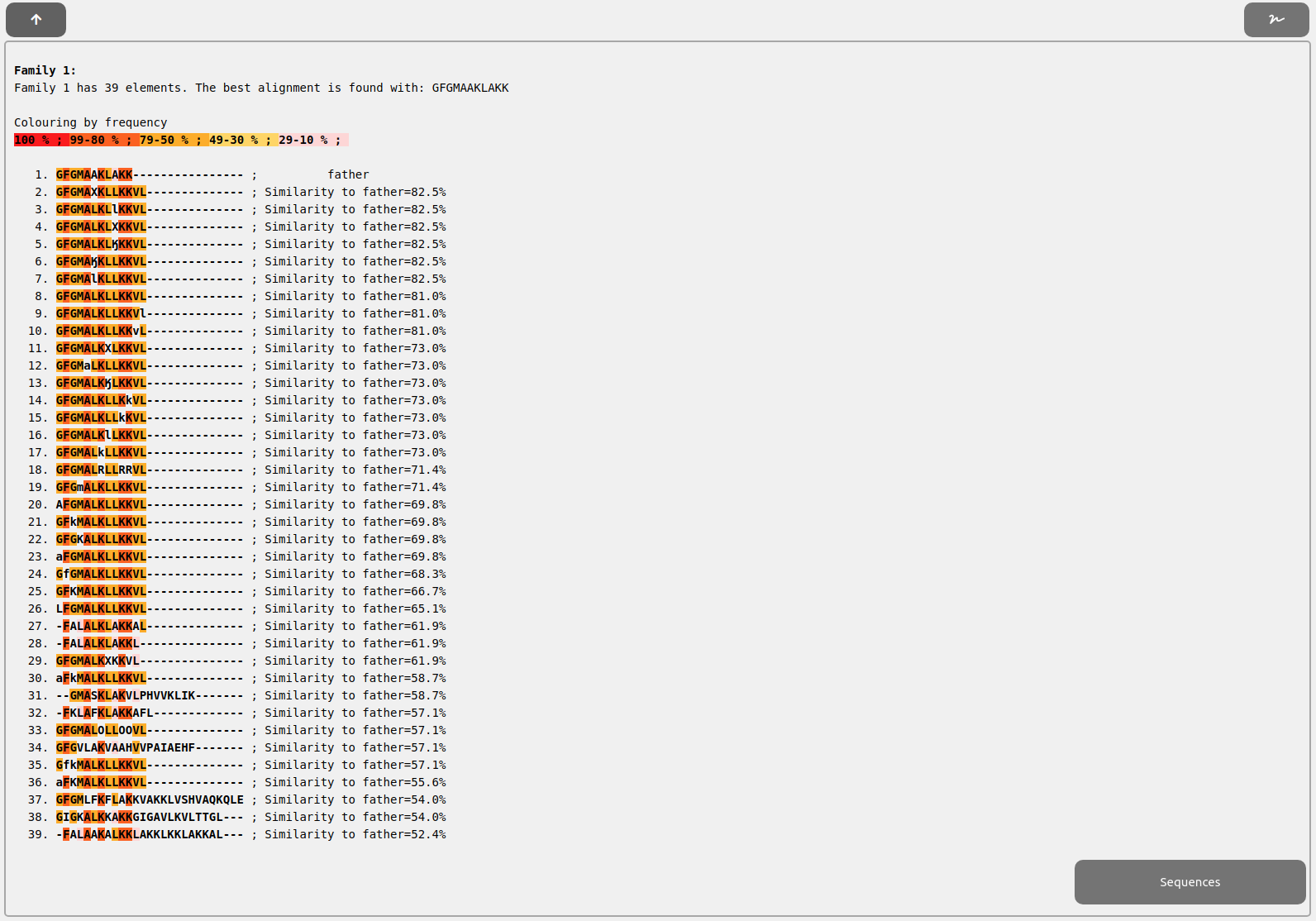


Our peptide (peptide 1) is quite similar to other peptides in the databases. Some of them (like peptide number 5 or 13) have sequences with non-standard aminoacids. This aminoacids are handled in the same way as regular aminoacids and represented by different characters to recognize them. To try to handle them more easily, we provide a button when necessarily next to the fields/sections that would need it (Screenshot 2.9). The button will open a section (Screenshot 2.10) allowing you to search for characters or aminoacids (pressing Ctrl+F), and to paste those characters in relevant fields when needed (simply clicking on them will paste the characters to the field next to the button used to open the character picker).

***Screenshot 2.9.***


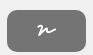


***Screenshot 2.10.***

***
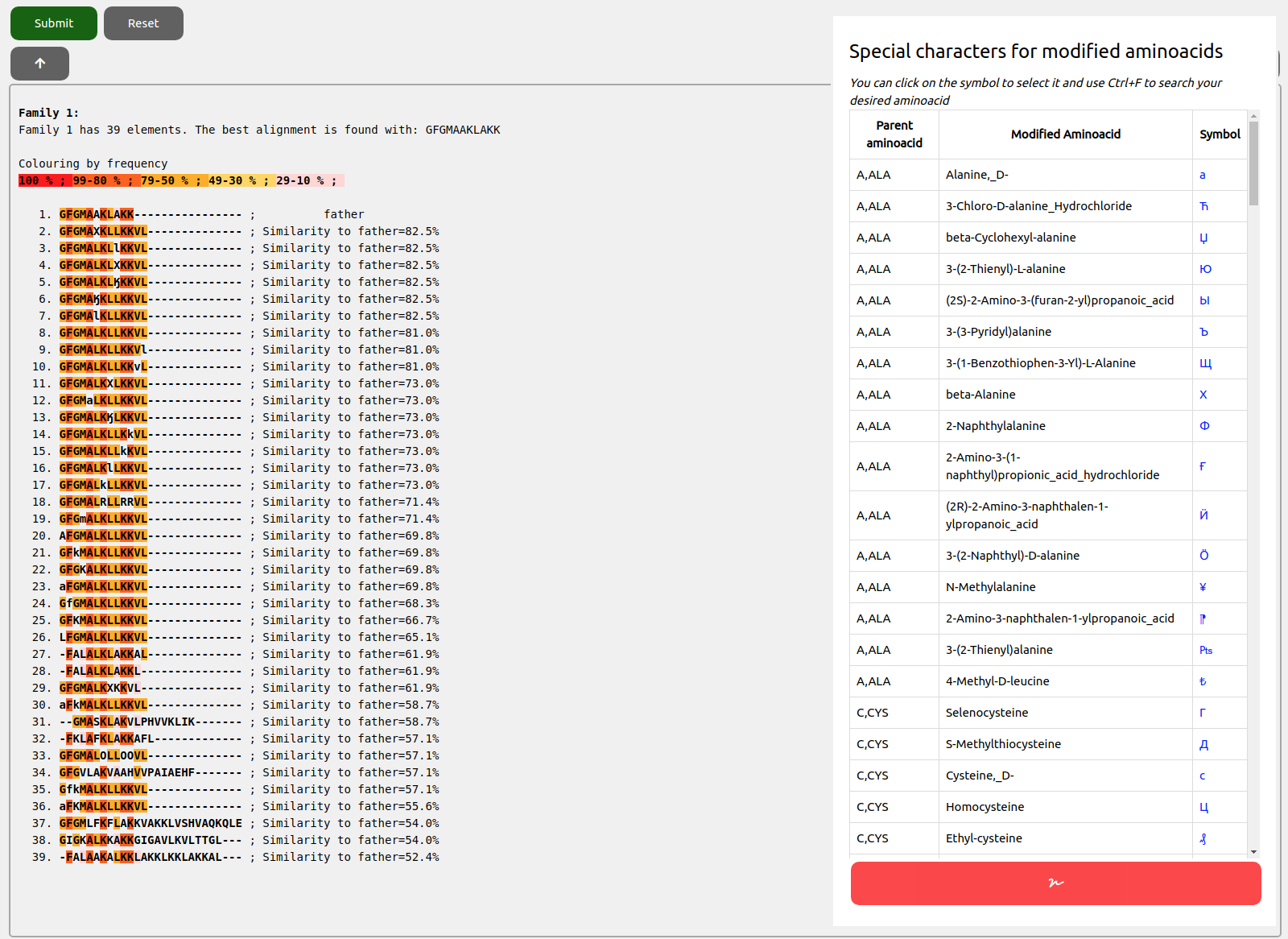
***

To find more information about the properties of the similar peptides we have several options: rely on FAMILY ANALYZER utilities to review each field we are interested in with full detail, click on “Available data per sequence” to show a table showing a summary or rely on the more readable table provided into the Full output html. We will opt for this last option and, to open it, we simply need to scroll up to “Global properties” section and click on “full output” link:

***Screenshot 2.11.***


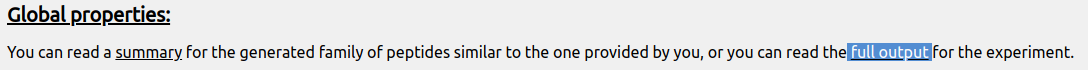


Then, we click into “Property details” and we will see the table.

***Screenshot 2.12.***


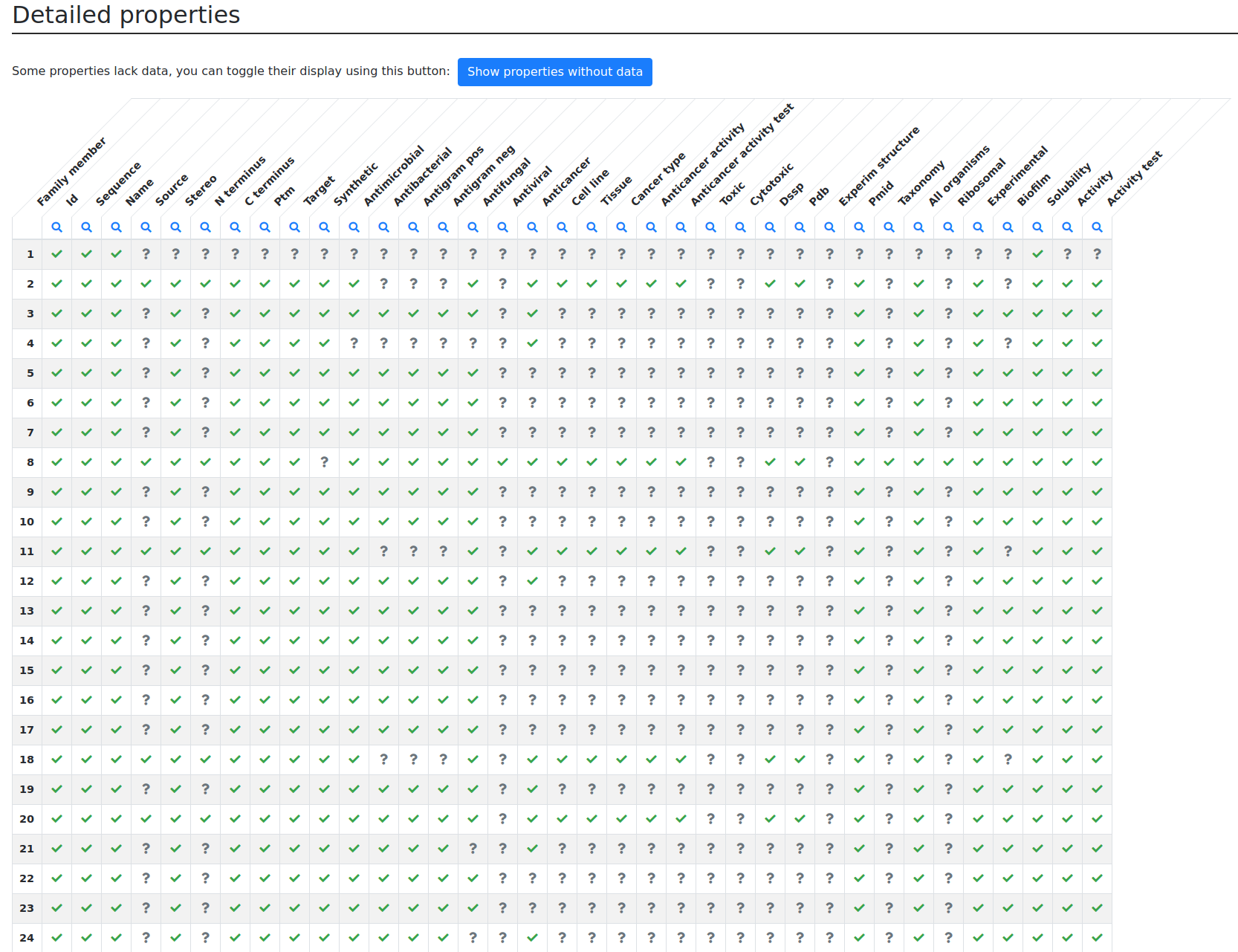


We discover that the peptides similar to our test sequence are antifungal and anticancer. In the list of similar peptides, the order follows the degree of similarity. This means that the most similar molecule is peptide 2, which is also antimicrobial. Given the sequence similarity we can predict that our peptide is likely to be antifungal and anticancer, possibly also antimicrobial then.

Looking into “Specific parameters” section, we can find more detailed information on a possible “cell_line”, including “tissue” and “cancer_type”.

***Screenshot 2.13.***

***
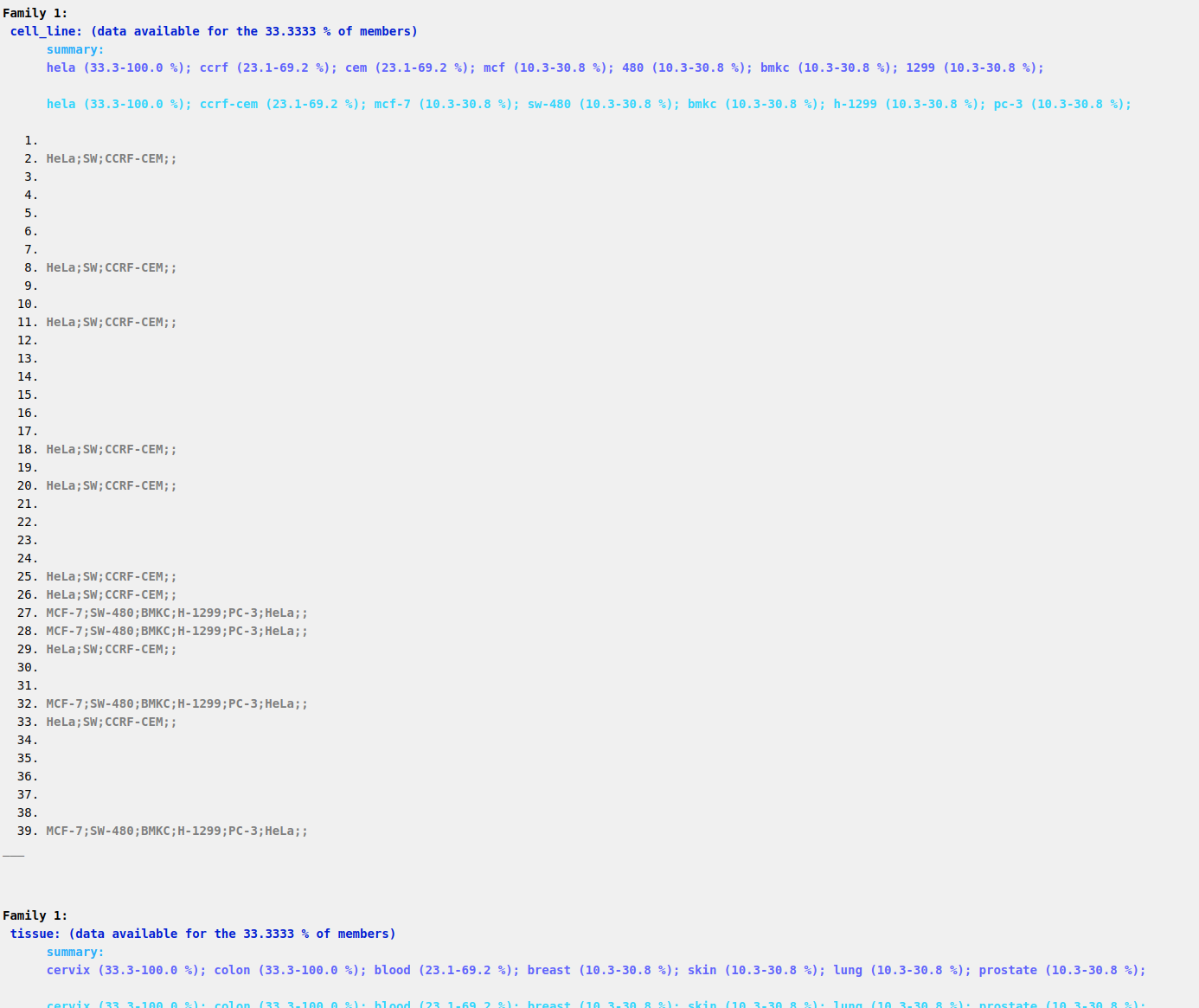
***

Following the list, we can predict the activity with decreasing accuracy. For example, our compound has relatively good chances to act on HeLa, SW and CCRF-CEM cell lines for Cervix, Colon and Lung cancers (properties of entry 2).

The output (section “Source”) also indicates that our compound can be considered part of the family of Macropins peptides:

***Screenshot 2.11.***

***
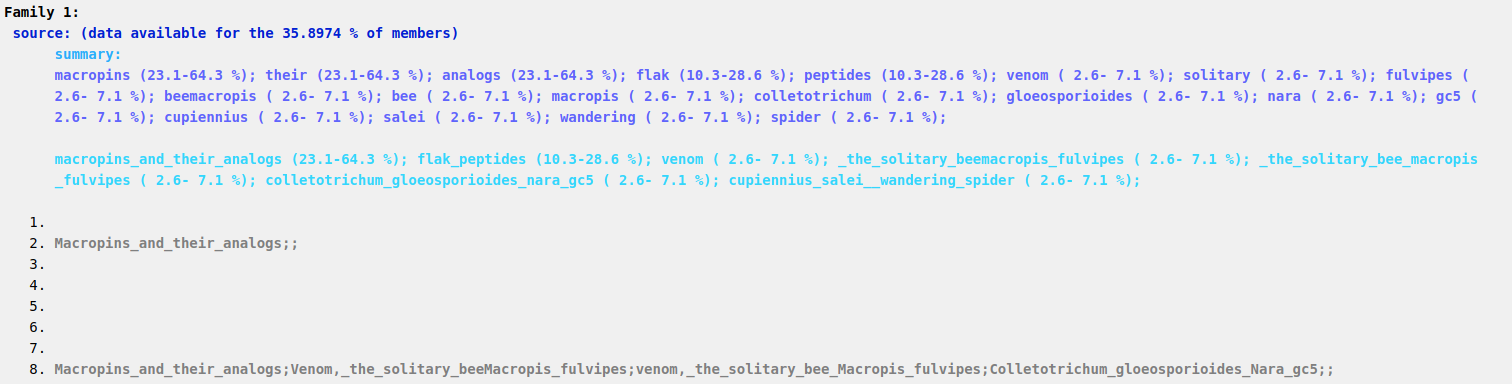
***

# ADAPTABLE TUTORIAL - CASE EXAMPLE n.3

## Discovering new activities of pre-existing sequences not tested experimentally

### The case of *Acinetobacter baumannii*

Despite the huge amount of experimental data on antimicrobial peptides, databases contain only a limited number of active peptides against some specific organisms. For example, when searching for peptides active against *A. baumannii*, if we look into peptides with an activity of not more than 0.01µM to kill the target organisms we obtain only 2 families formed by only 1 aminoacid each.

***Screenshot 3.1.***


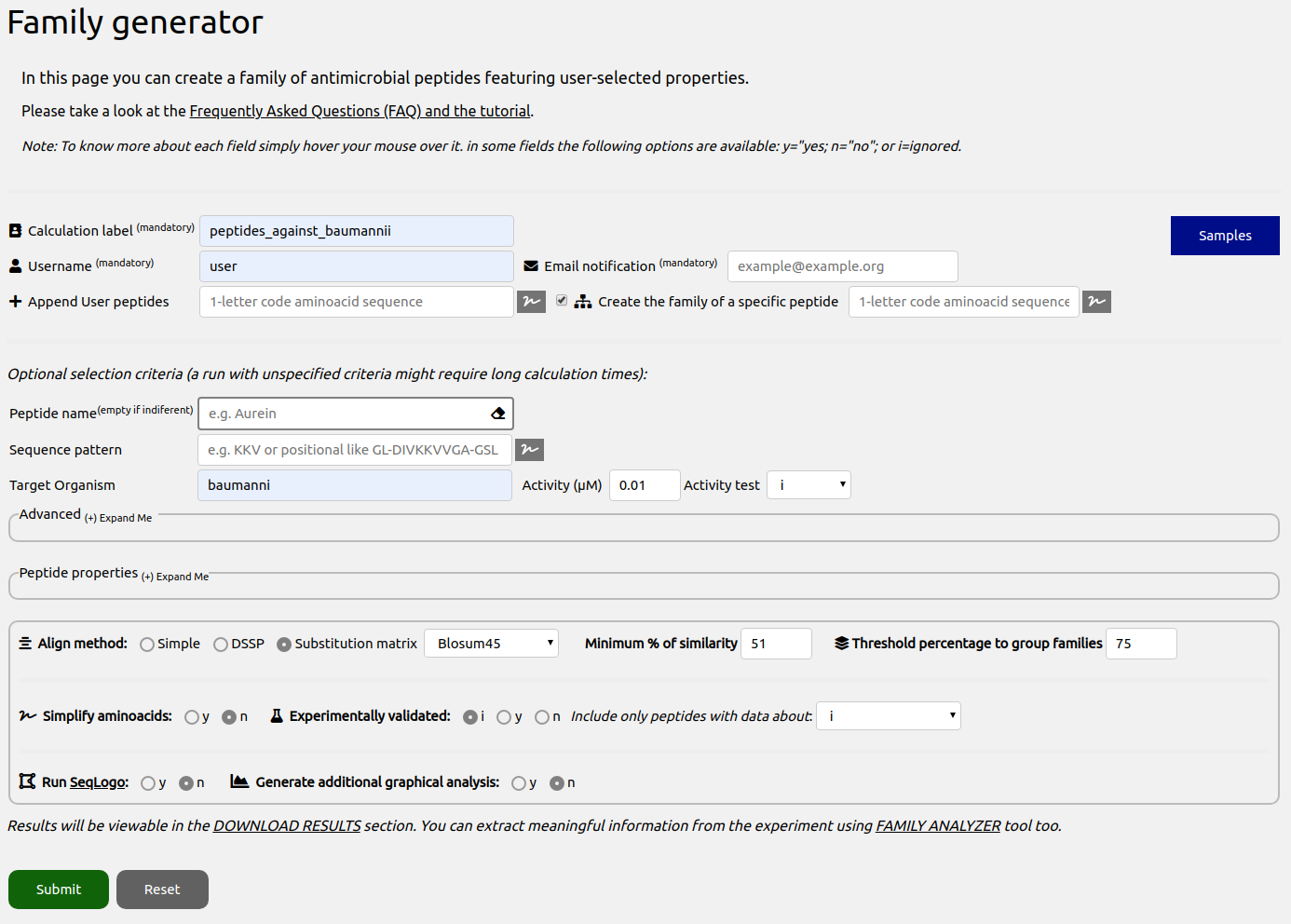


*A. baumannii* has been declared among the most dangerous pathogen in terms of antibiotic resistance by the World Health Organization (WHO). It is therefore very important to find new active compounds which can be used as drugs. To find new peptides that could be similar to this two ADAPTABLE can help in this task. For example, focalizing on peptide 1 (GWLKKIGKKIERVGQHTRDATIQTIGVAQQAANVAATLK), we can use the “Create the family of a specific peptide” feature (see details in the previous example) to find interesting peptides. It allows to compare a given sequence (in our case GWLKKIGKKIERVGQHTRDATIQTIGVAQQAANVAATLK), with our full AMPs database. When running the calculation we obtain 31 potentially active peptides which were never tested with *A. Baumannii* as can be seen in the summary page that is offered shown after the experiment run. That page summarizes the properties (*screenshot 3.2*) and lists the members (*screenshot 3.3*) of the family of peptides similar to GWLKKIGKKIERVGQHTRDATIQTIGVAQQAANVAATLK.

***
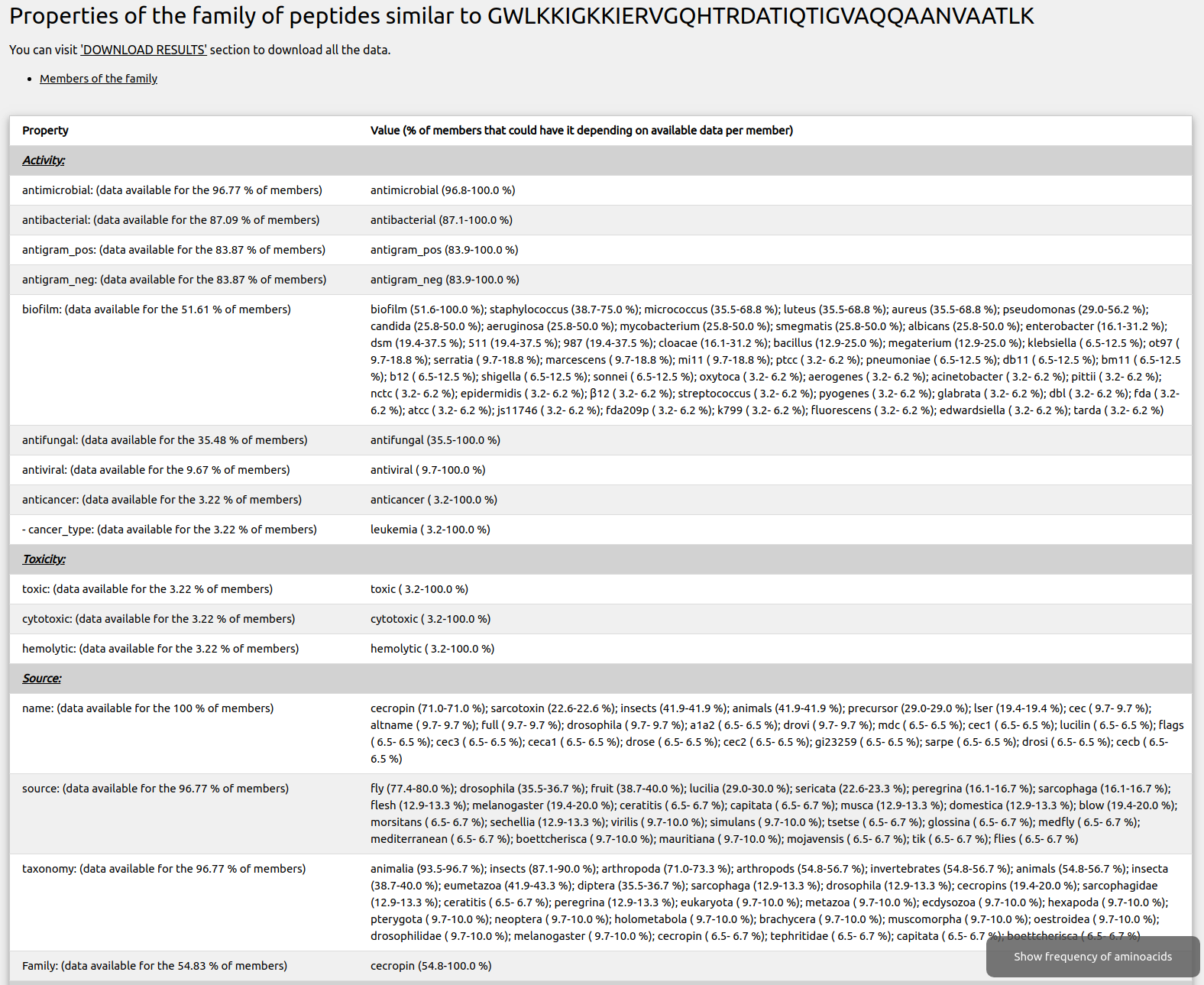
Screenshot 3.2.***

***
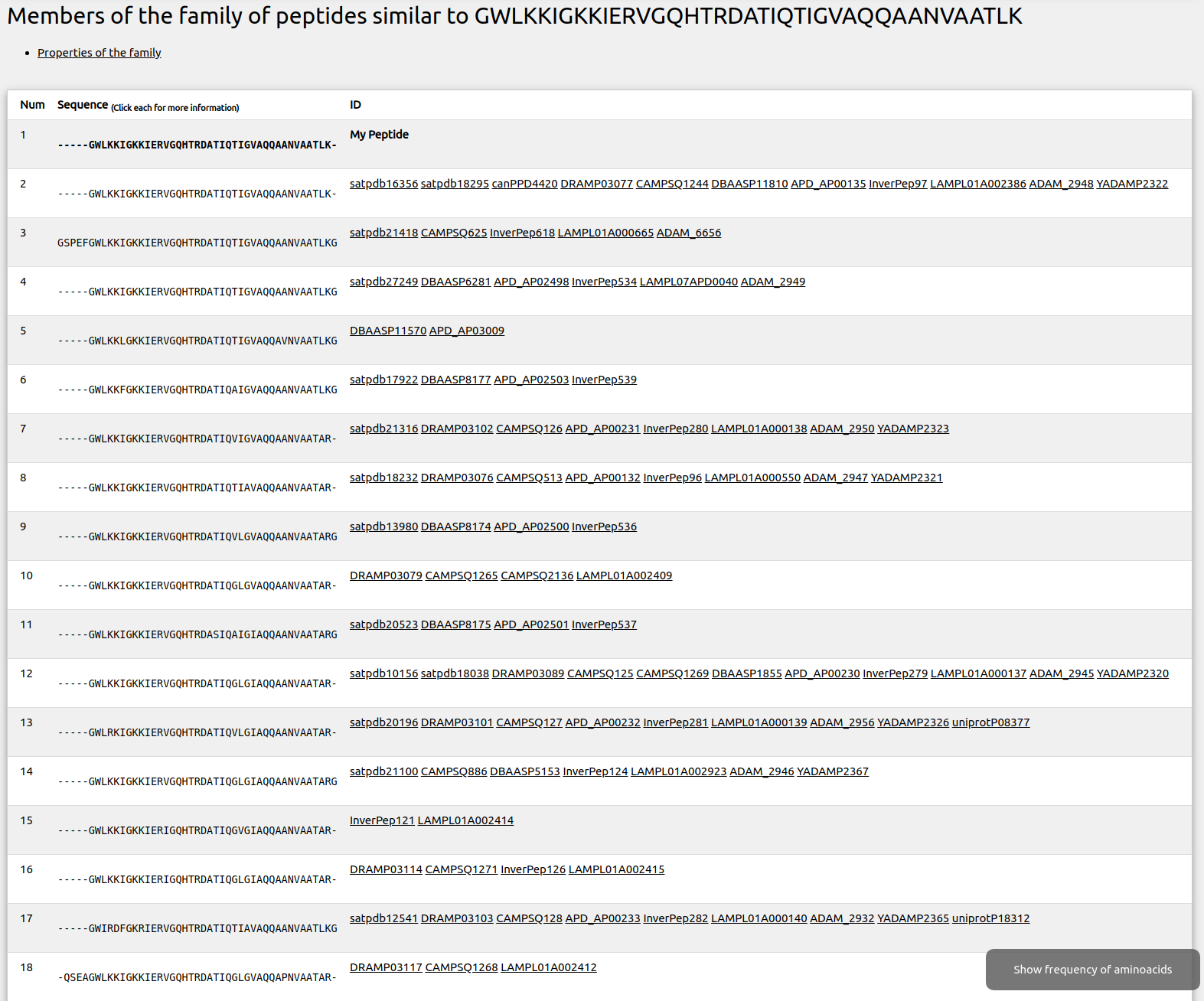
Screenshot 3.5.***

A quick look to the data table (“Available data per sequence”) reveals that some of the new potentially active peptides are not hemolytic. In the table, each line corresponds to one peptide and each column corresponds to a property (1 indicates presence of the property). A “1” in column 37 indicates that the peptide has been tested experimentally for its hemolytic activity, while column 38 expresses whether the peptide is hemolytic (value 1) or not (value 0). In the former case, the intensity of the blue color indicates the extent of the hemolytic activity (**(sub-μM)-(μM)-(sub-mM)-(mM)-(very weak)**).

This way we can select peptides with activities we want or activities we want to skip (like toxic or hemolytic activities).

***Screenshot 3.7.***

***
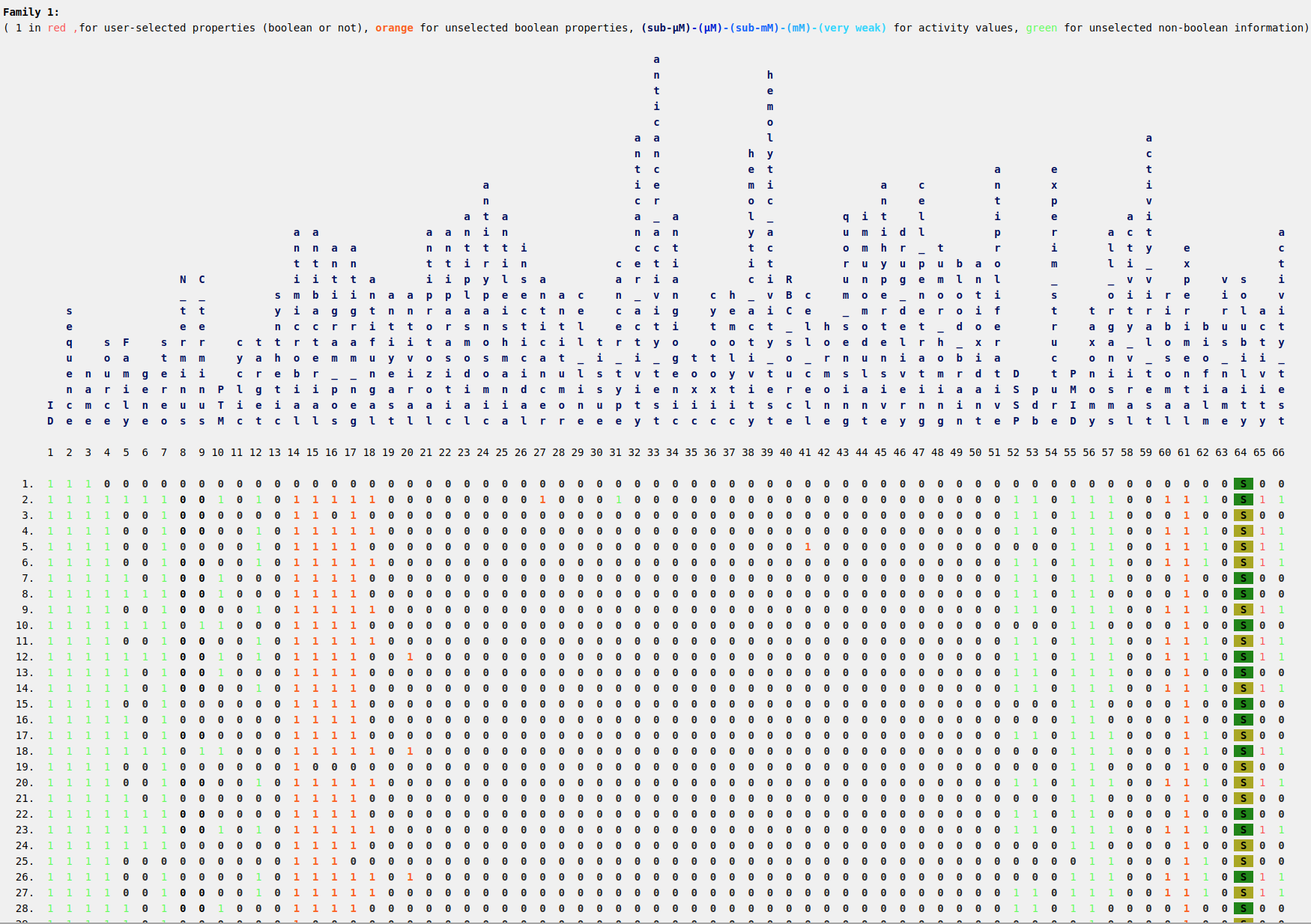
***
